# Rigid Residue Scan Simulations Systematically Reveal Residue Entropic Roles in Protein Allostery

**DOI:** 10.1371/journal.pcbi.1004893

**Published:** 2016-04-26

**Authors:** Robert Kalescky, Hongyu Zhou, Jin Liu, Peng Tao

**Affiliations:** 1 Department of Chemistry, Center for Drug Discovery, Design, and Delivery (CD4), Center for Scientific Computation, Southern Methodist University, Dallas, Texas, United States of America; 2 Department of Pharmaceutical Sciences, University of North Texas System College of Pharmacy, University of North Texas Health Science Center, Fort Worth, Texas, United States of America; Wichita State University, UNITED STATES

## Abstract

Intra-protein information is transmitted over distances via allosteric processes. This ubiquitous protein process allows for protein function changes due to ligand binding events. Understanding protein allostery is essential to understanding protein functions. In this study, allostery in the second PDZ domain (PDZ2) in the human PTP1E protein is examined as model system to advance a recently developed rigid residue scan method combining with configurational entropy calculation and principal component analysis. The contributions from individual residues to whole-protein dynamics and allostery were systematically assessed via rigid body simulations of both unbound and ligand-bound states of the protein. The entropic contributions of individual residues to whole-protein dynamics were evaluated based on covariance-based correlation analysis of all simulations. The changes of overall protein entropy when individual residues being held rigid support that the rigidity/flexibility equilibrium in protein structure is governed by the La Châtelier’s principle of chemical equilibrium. Key residues of PDZ2 allostery were identified with good agreement with NMR studies of the same protein bound to the same peptide. On the other hand, the change of entropic contribution from each residue upon perturbation revealed intrinsic differences among all the residues. The quasi-harmonic and principal component analyses of simulations without rigid residue perturbation showed a coherent allosteric mode from unbound and bound states, respectively. The projection of simulations with rigid residue perturbation onto coherent allosteric modes demonstrated the intrinsic shifting of ensemble distributions supporting the population-shift theory of protein allostery. Overall, the study presented here provides a robust and systematic approach to estimate the contribution of individual residue internal motion to overall protein dynamics and allostery.

## Author Summary

Allostery is a fundamental dynamics property of many proteins, and plays a critical role in protein functions. Despite extensive experimental and theoretical studies of protein allosteric mechanisms, the current understanding and predicting power of protein allostery are still limited. One of the main challenges in studying protein allostery is effectively narrowing down residues for further site-directed mutagenesis study. Our goal is to develop effective computational tools to systematically evaluate significance of individual residue in protein dynamics and allostery without any *a priori* knowledge about protein allosteric mechanism. In this study, we significantly enhanced a simulation protocol developed in our lab, rigid residue scan (RRS), through combination of configurational entropy calculation, principal component analysis (PCA), and projection of ensembles onto coherent allosteric modes. Detailed analysis of the impact of removing individual residue internal motions on overall protein dynamics led to identification of key allosteric residues. Our prediction of key allosteric residues has good agreement with experimental studies of an allosteric protein as a model system, which displays allostery through binding events. Interestingly, the entropy calculations suggest that the La Châtelier’s principle in chemical equilibrium may also govern the rigidity/flexibility equilibrium in protein structure, which is related to protein allostery. Our study has demonstrated these methods to be very valuable tools to effectively identify initial key residues for proteins with crystallographic structures and limited information of their allosteric mechanisms.

## Introduction

Allostery is the process by which signals are transmitted from distal ligand binding sites to functional sites in proteins. The concept of allostery originated from early attempts to explain the fact that the binding of oxygen molecules to hemoglobin deviates from the typical Michaelis-Menten kinetics model.[1–3] Following the term “allosteric” being coined and reviewed during early 60’s,[4, 5] two protein allostery theories were proposed and referred to as the Monod−Wyman−Changeux (MWC)[6] and Koshland−Neḿethy−Filmer (KNF)[7] models. In these models, allostery theories were formed based on significant conformational changes of hemoglobin observed in crystallographic structures. In addition to hemoglobin, allostery with conformational change has been observed in other proteins such as aspartate transcarbamoylase,[8] insulin,[9] trypsin,[10] and caspases[11]. In these proteins, the binding signal is assumed to be transmitted through protein conformational change.

Multiple allostery theories have evolved based on experimental and theoretical studies.[12–27] The classical “induced fit” model[28–30] fits well to protein conformational changes upon ligand binding observed in hemoglobin.[31] However, a more recent “population shift” model of protein allostery[32–35] is strongly supported by sophisticated NMR experiments.[36–38] In this model, no conformational changes can be detected throughout the process in which proteins carry out their functions. Instead, allostery-triggering events alter the distribution of the protein ensemble among distinctive sub-states.

Many computational methods have been developed to delineate protein allosteric mechanisms in atomic detail and to facilitate development of allostery theories. Some methods are mainly based on protein tertiary structure comparison using topology or graph theory for analysis.[39–45] Some methods analyze energy-based residue-residue interactions to explore residue coupling.[46–50] Normal mode analysis (NMA)[51] is employed based on the elastic network model (ENM)[52, 53] or the Gaussian network model (GNM).[54] These models provide coarse-grained protein structure descriptions, which reduce the computational cost to probe the protein’s vibrational modes. Modes with low frequency and large magnitude presumably correspond to allosteric mechanisms.[55–58] Molecular dynamics (MD) simulation is the most widely used and direct means to simulate protein dynamics. Thus, it is frequently used with certain modifications to investigate protein allostery as a dynamical process. Ota and Agard proposed the MD-based anisotropic thermal diffusion (ATD) method to probe energy dissipation pathways in proteins. In this method, a single residue is heated in a protein at an extremely low temperature (approximately 10K) to probe energy dissipation pathways.[59] Sharp and Skinner developed a pump-probe MD method that perturbs protein dynamics by exerting oscillating forces on target residues.[60] Long-time MD simulations were carried out and subjected for further analysis to reveal protein allosteric effects in several other studies.[35, 61–64]

Deep understanding of protein allostery remains elusive despite the experimental and theoretical studies done thus far. More methodological development is needed to quantitatively evaluate the effect of individual amino acid residues on overall protein dynamics. Although mutagenesis studies can provide valuable information about the impact of changing specific residues on protein activity, systematically posing perturbation on individual residues provides an alternative way to probe the effect of the internal motions of specific residues on protein dynamics or to discover the function of individual residues without changing their chemical entity. Applying rigid constraints on selected degrees of freedom in protein structure has been implemented to probe protein allostery.[65–67] Alternatively, we recently developed a simulation method, referred to as rigid residue scan (RRS),[68] to systematically probe the impact of each individual residue on overall protein dynamics through rigid body MD simulations using an efficient integrator.[69] In this study, the entropy calculation and principal component analysis are combined with the RRS method to evaluate the effects of internal motions from individual residues on overall protein dynamics as well as allostery upon ligand binding.

## Results

### Root-Mean-Square Deviation (RMSD) and Average Structures from Simulations

**Fig 1:**
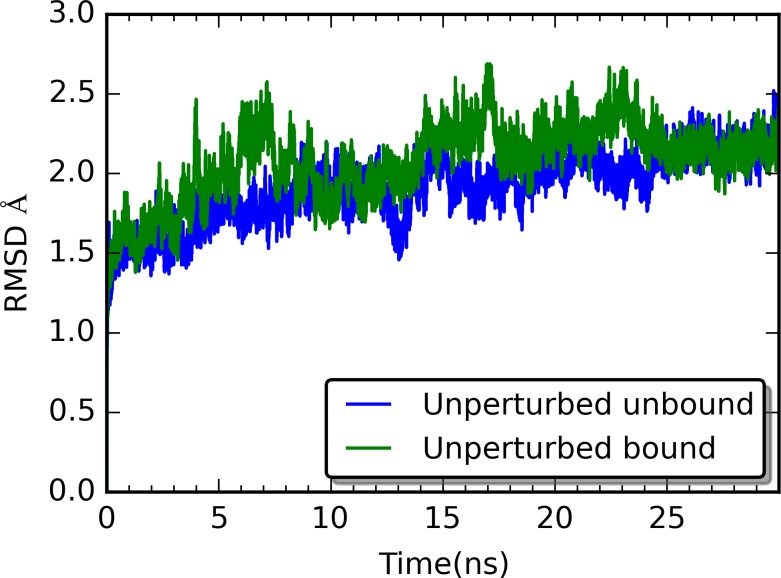
RMSD for the unperturbed (no rigid residue perturbation) molecular dynamics simulations of both unbound and bound PDZ2. The RMSD is determined relative to the initial simulation structure of each simulation.

**Fig 2:**
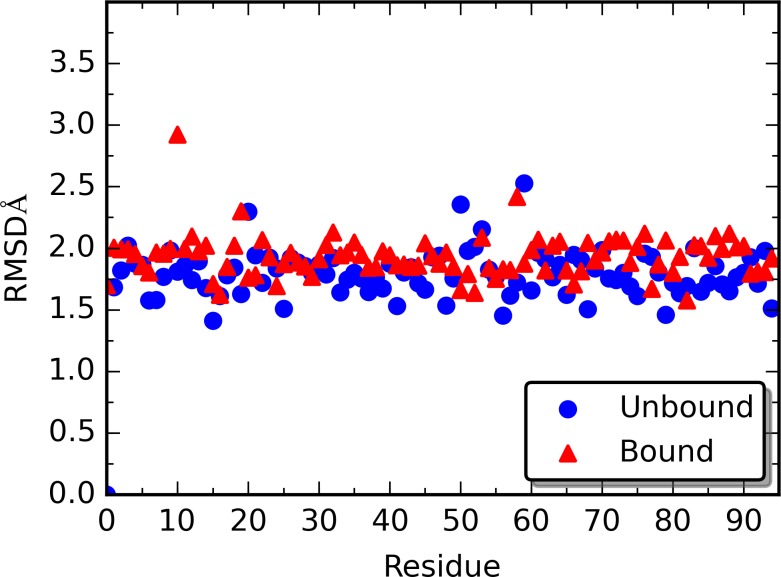
All-atom RMSD of average structures of 190 RRS MD simulations.

The all-atom RMSD for the unperturbed unbound and bound states (without rigid residue perturbation) of PDZ2 are shown in Fig 1, which indicates that both structures are stable throughout the simulations. The RMSD plots of all RRS simulations are listed in S1 Table. In general, the RRS simulations are stable throughout the simulations, with the majority of the simulations having average RMSD under 2 Å (Fig 2).

To assess the overall conformational change upon binding and rigid residue perturbation, averaged structures of PDZ2 were calculated for each simulation. Using the averaged structure of PDZ2 from unperturbed unbound simulation as reference structure, the all-atom RMSD of all other averaged structures were calculated and plotted in Fig 2. For most of the RRS simulations, both unbound and bound, the average structures have RMSD between 1.5 and 2.0 Å, with very few exceptions.

### Changes of Protein Entropy upon Perturbation

**Fig 3:**
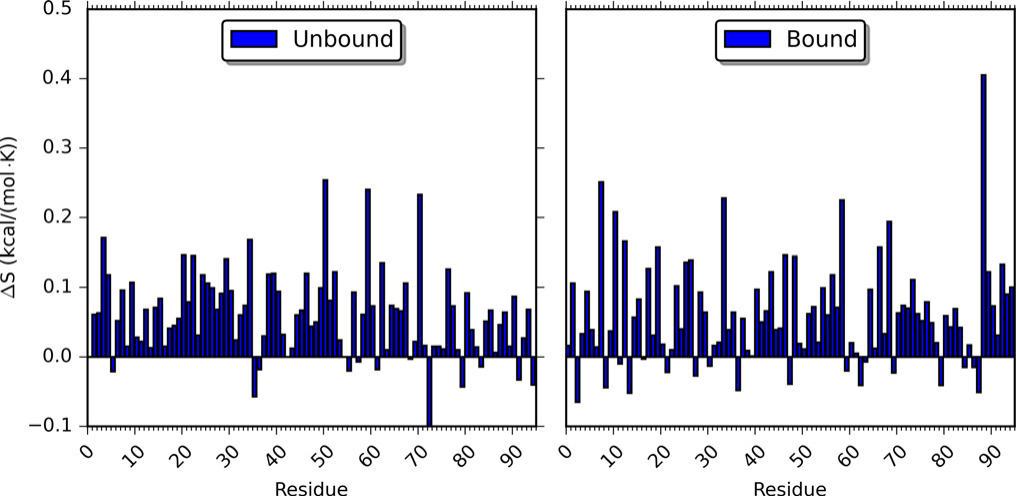
Relative entropy (ΔS) of PDZ2 in unbound and bound states from RRS simulations. The entropy of PDZ2 from unperturbed simulations is used as reference.

Entropy contributions from PDZ2 are estimated using the method[70, 71] described in the Computational Methods section for all the unperturbed and rigid body perturbed simulations. The heat maps of cross-correlation matrices (based on which the entropy was calculated) are provided for all the simulations in S2 Table. Using the entropy of PDZ2 from unperturbed simulation of unbound state as reference, the relative entropies of PDZ2 (ΔS) from all the simulations are plotted in Fig 3 and listed in S3 Table. The ΔS are also sorted with ascending order and listed in S4 Table.

**Fig 4:**
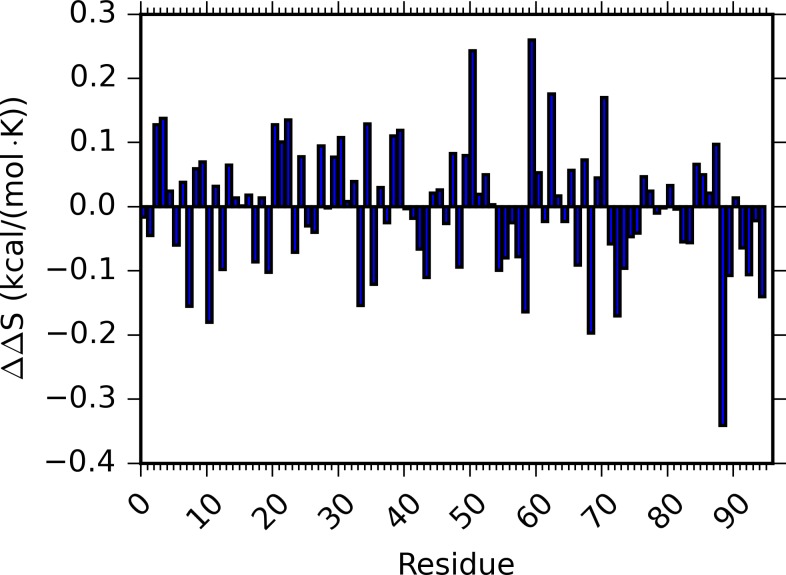
Entropy difference (ΔΔS) between unbound and bound PDZ2 states from unperturbed and RRS simulations.

For both unbound and bound states, the entropy of PDZ2 significantly increases for most of the RRS simulations. The changes of unbound PDZ2 entropy in rigid residue simulations comparing to unperturbed simulations vary from −0.100 to 0.254 kcal/(mol•K) with average as 0.058 kcal/(mol•K) and unsigned average as 0.066 kcal/(mol•K). For the bound state, the PDZ2 entropy differences in rigid residue simulations comparing to unperturbed simulation vary from −0.065 to 0.405 kcal/(mol•K) with average as 0.060 kcal/(mol•K) and unsigned average as 0.071 kcal/(mol•K). Overall, in 80 unbound and 68 bound RRS simulations, the ΔS of PDZ2 is positive. This is counterintuitive, because treating a residue as a rigid body removes the internal degrees of freedom of that residue and should reduce the overall entropy. Furthermore, we calculated the PDZ2 entropy difference between two states (ΔΔS) by subtracting the unbound state entropy from the bound state entropy with the same residue being held rigid (Fig 4 and S3 and S4 Tables). The absolute ΔΔS values range from 0.001 to 0.341 kcal/(mol•K) (S4 Table). For the two unperturbed simulations, this difference is 0.016 kcal/(mol•K) with a higher PDZ2 entropy from the bound state (Residue 0 in S3 and S4 Tables). For the 11 residues being held rigid, the absolute ΔΔS is smaller than 0.016 kcal/(mol•K) (S4 Table). Seven among these 11 residues, D15, T28, V40, T81, R31, L78, L18, were reported as important allosteric residues from an NMR study of PDZ2 bound to the RA-GEF-2 peptide (Table 1).[72]

**Table 1:** Key residues for PDZ2 allostery upon RA-GEF2 peptide binding from NMR study [72].

**D15**,^a^ **N16**, **L18**, I20, **V22**, **V26**, **N27**, **T28**, V30, **R31**, G34, A39, **V40**, **V61**, V64, L66, A69, H71, **L78**, **T81**, **V85**

^a^ Residues in bold type are recognized from the present study.

The error bar of PDZ2 entropy calculations was estimated for both unbound and bound states of PDZ2 in unperturbed simulations and seven RRS simulations (Table 2). For these simulations, the PDZ2 entropy was calculated based on seven sets of 30 ns (total of 210 ns) trajectories. The standard deviation (σ) and 85% Confidence Interval (CI) of each state is rather small, indicating the convergence of simulation within 30 ns of trajectories. The standard deviation (σ) and 85% CI of ΔΔS for unperturbed states are 0.037 and 0.039 kcal/(mol•K), respectively. It is noticeable that the errors of entropy calculations, although small when compared to the total entropy, are comparable to the differences between simulations. For the unbound state, total of 28 RRS simulations have ΔS smaller than the 85% CI of unperturbed unbound state. For the bound state, total of 53 RRS simulations have ΔS smaller than the corresponding 85% CI (S3 Table). Among seven identified residues, there are three residues (D15, T28, V40) with ΔS values higher than the 85% CI of corresponding unperturbed states. Although the cancelling of the error could improve the reliability of the analysis, these comparisons indicate that the uncertainty of calculated configurational entropies requires further improvement, for example by including anharmonicity and higher order correlations, to increase the reliability of the calculations.

**Table 2:** Estimation of error bar for PDZ2 entropy calculations (kcal/(mol•K))^a^.

Rigid	Average S (σ, 85% Confidence Interval)	
Residue	Unbound	Bound
none	5.202 (0.046, 0.029)	5.238 (0.098, 0.061)
15	5.213 (0.086, 0.054)	5.241 (0.068, 0.042)
18	5.154 (0.045, 0.028)	5.224 (0.047, 0.029)
22	5.208 (0.058, 0.036)	5.203 (0.056, 0.035)
28	5.180 (0.038, 0.024)	5.185 (0.059, 0.037)
40	5.269 (0.064, 0.040)	5.183 (0.033, 0.021)
61	5.206 (0.084, 0.052)	5.250 (0.079, 0.049)
81	5.208 (0.039. 0.024)	5.237 (0.073, 0.046)

^a^ For each state, seven sets of 30 ns trajectories were used.

Velocity autocorrelation analysis was carried out for the unperturbed simulations and the seven RRS simulations listed in Table 2 to estimate the relaxation time in these simulations. Only one trajectory of each simulation was subjected to the analysis. All the selected simulations display a relaxation time around 20 ps (S1 Fig), showing that RRS simulations have similar relaxation time to the unperturbed simulations.

### Individual Residue Response upon Perturbation

**Fig 5:**
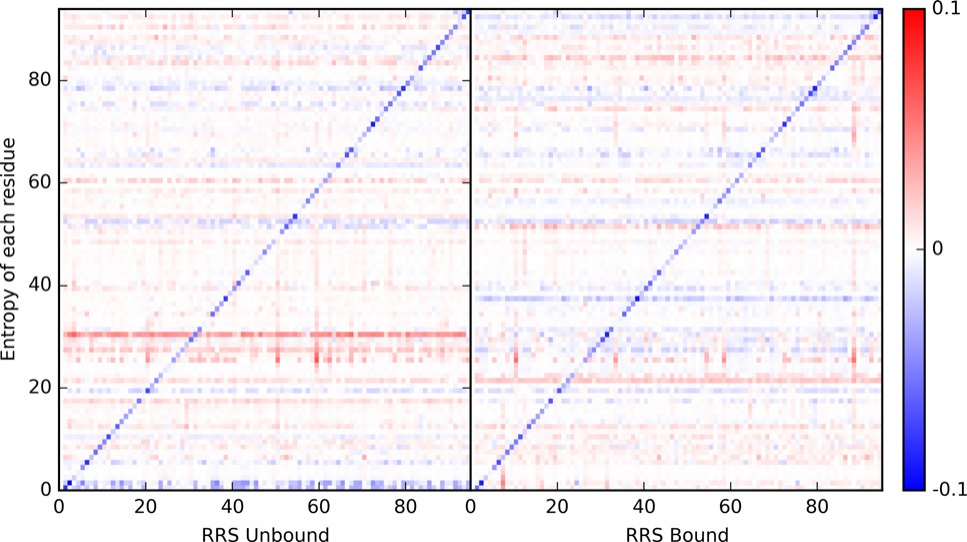
Heat maps of individual residue entropic contribution under rigid residue perturbation for unbound (left) and bound (right) states. The entropic contribution from each residue in unperturbed simulations (with index as 0 in both plots) is set as reference.

**Fig 6:**
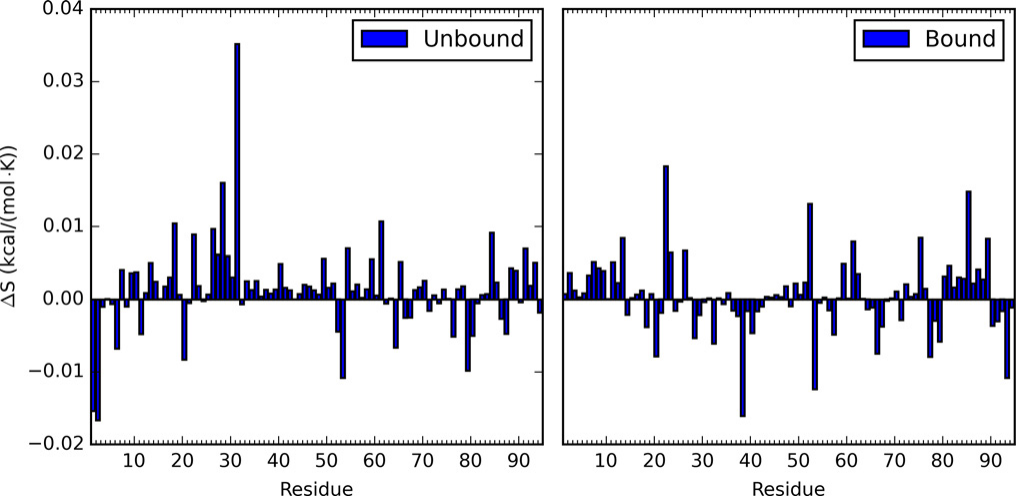
Average entropic response from each residue in all RRS simulations.

From each simulation, the entropy contribution of each residue to total protein entropy was evaluated. Such individual residue entropy contributions are plotted as heat maps for the RRS simulations of both unbound and bound states of PDZ2 (Fig 5). To make plots clear, the contribution from each individual residue in unperturbed simulations was used as reference in unbound and bound states, respectively. The response of each individual residue varies significantly. The most prominent features in both heat maps are the blue diagonal lines, reflecting the fact that the entropy contribution from each residue diminishes when that residue is held as a rigid body during the simulation. The most recognizable features besides blue diagonal lines are the horizontal lines in both heat maps, either in red or blue. These horizontal red or blue lines indicate that response from some residues to rigid body perturbation is consistent regardless which residue being held rigid in the perturbed simulations. To further illustrate this feature, the average entropic response from each residue in all RRS simulations was calculated and plotted in Fig 6 for the unbound and bound states, respectively. The average entropic responses are also listed in S5 Table and with descending order in S6 Table. The individual residue entropies were also normalized using the number of atoms in each residue following a previous study.[73] The normalized individual residue entropies are illustrated in S2 Fig and S3 Fig. The patterns described above remain the same with the normalized entropies, showing that the differences of residue responses are inherent to each residue and not scaled with residue size.

The average entropic responses range between 0.0352 and −0.0167 kcal/(mol•K) for the unbound state, and between 0.0183 and −0.0161 kcal/(mol•K) for the bound state. Among the top ten residues with largest average entropic responses in the unbound state, seven residues, R31, T28, V61, L18, V26, V22, N27, were among the 21 important allosteric residues reported in an NMR study of PDZ2 (Table 1).[72] However, for the bound state, only four residues, V22, V85, V61, V26, among top ten residues were reported as important allosteric residues from the same NMR study of PDZ2 (Table 1).[72] Noticeably, three residues, V22, V61, and V26, are among the top ten residues of both unbound and bound states.

### Quasi-harmonic Analysis and Principal Component Analysis (PCA)

**Fig 7:**
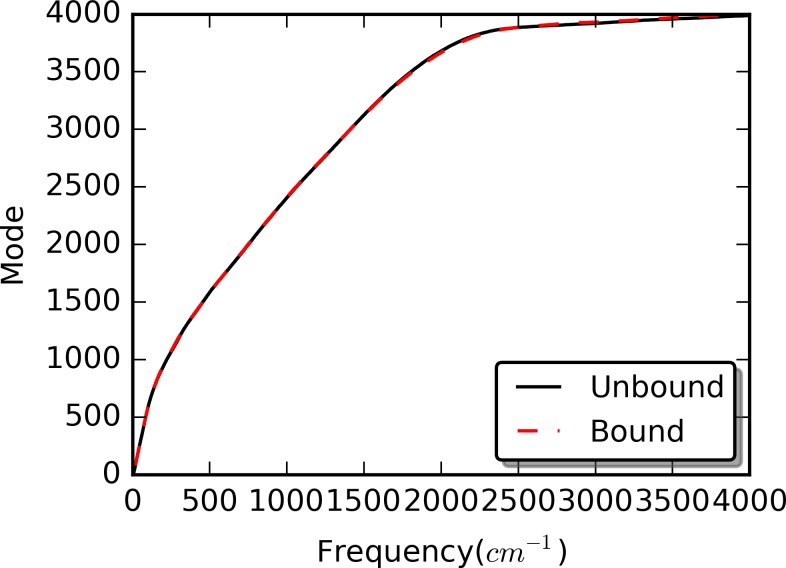
Distributions of density of states for unperturbed unbound and bound states.

**Fig 8:**
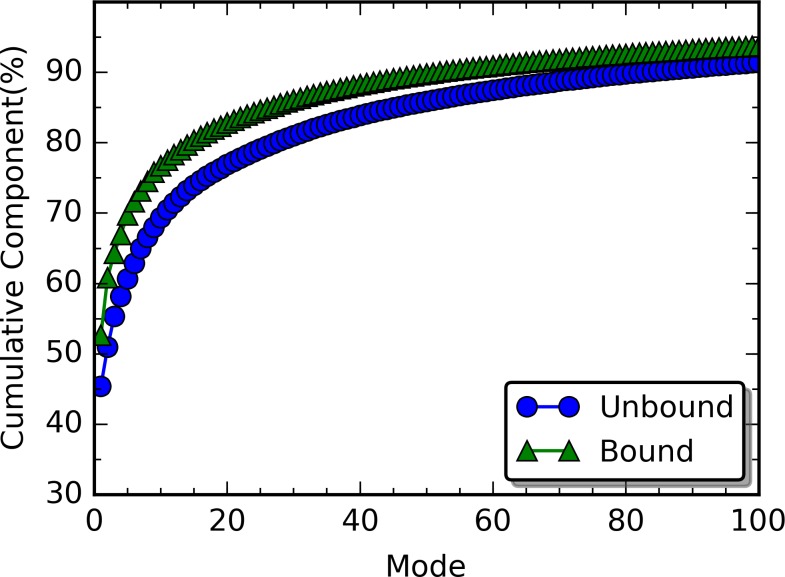
PCA contributions in unperturbed unbound and bound states.

Quasi-harmonic analysis was carried out for both unperturbed and RRS simulations. The distributions of density of states from quasi-harmonic analysis of unperturbed simulations are plotted for both unbound and bound states in Fig 7. Obviously, the binding with the peptide does not significantly affect the distribution of density of states. Similarly, the rigid residue perturbation does not significantly affect the distribution of density of states either (S4 Fig). We further carried out the PCA to evaluate the contribution of each quasi-harmonic mode to overall dynamics, and plotted accumulative contribution of these modes for both unperturbed unbound and bound states in Fig 8. Low frequency modes significantly contribute to overall protein dynamics. For the unbound state, total of 83 modes with frequency under 18.7 cm^−1^ contribute 90% to the overall dynamics. For the bound state, total of 52 modes with frequency under 13.4 cm^−1^ contribute 90% to the overall dynamics. In both cases, translational and rotational modes are excluded. Because protein allostery is highly dynamical process that couples the dynamics of distal parts of the protein, it is logical to assume that mainly low frequency modes, which involve overall dynamics of proteins, play important roles in protein allostery.

To identify significant low-frequency modes, PCA was carried out for the seven 30 ns trajectories as well as total 210 ns trajectories for the unperturbed unbound and bound states, respectively. For the unperturbed unbound state, the dot products were calculated between the five lowest frequency quasi-harmonic modes (PC1 to PC5) of each 30 ns trajectory with the PC1 to PC5 modes from the whole 210 ns trajectory (S7 Table). The same calculations were also carried out for the unperturbed bound state (S7 Table). The unsigned averaged dot product of each mode is listed in Table 3. Among five modes, only PC1 modes (with the lowest frequency quasi-harmonic mode) in both unperturbed unbound and bound states have significant overlap between each trajectory and overall trajectory. The overlaps for PC2 through PC5 are significantly less than PC1 (Table 3), indicating that these modes and all other modes with higher frequencies do not have physical significance.

**Table 3:** Unsigned average dot products of five lowest frequency quasi-harmonic modes (PC1 through 5) between each 30 ns trajectory and whole 210 ns trajectory.

Modes	Average dot produces (σ)	
	Unbound	Bound
PC1	0.929 (0.044)	0.899 (0.051)
PC2	0.422 (0.171)	0.459 (0.227)
PC3	0.193 (0.116)	0.204 (0.150)
PC4	0.206 (0.128)	0.176 (0.103)
PC5	0.287 (0.177)	0.254 (0.175)

To further evaluate significance of PC1 modes in the unperturbed unbound state, the dot products among PC1 modes from seven 30 ns trajectories were calculated to produce a 7×7 matrix (S8 Table). The absolute values of off-diagonal matrix elements range from 0.694 to 0.960 with unsigned average values (standard deviation) as 0.842 (0.078). The similar analysis of the unperturbed bound state results in a matrix with absolute values of off-diagonal matrix elements range from 0.653 to 0.938 with average values (standard deviation) as 0.781 (0.089) (S8 Table). It should be noted that PC1 modes from 210 ns trajectories of unperturbed unbound and bound states do not overlap significantly with each other (with magnitude of dot product as −0.214). Therefore, two PC1 modes from two states could serve as coherent allosteric modes revealing effect of PDZ2 upon ligand binding. The PC1 modes calculated using 210 ns trajectories of the unperturbed unbound and bound states are used for further analysis in this study.

### Ensemble Distributions

**Fig 9:**
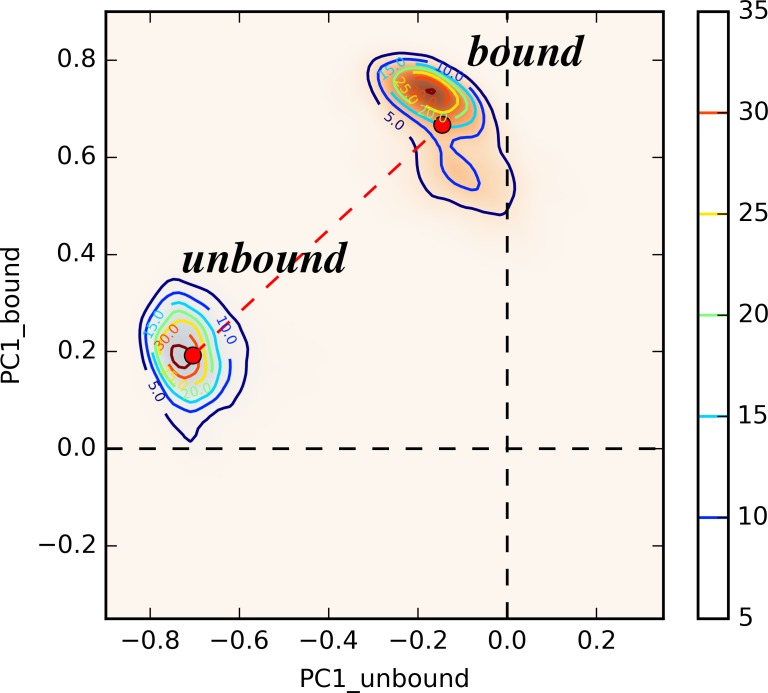
Distribution of unperturbed states projected onto a 2D surface using two PC1 modes. Only one set of 30 ns trajectories are used for sake of consistency with RRS simulations.

**Fig 10:**
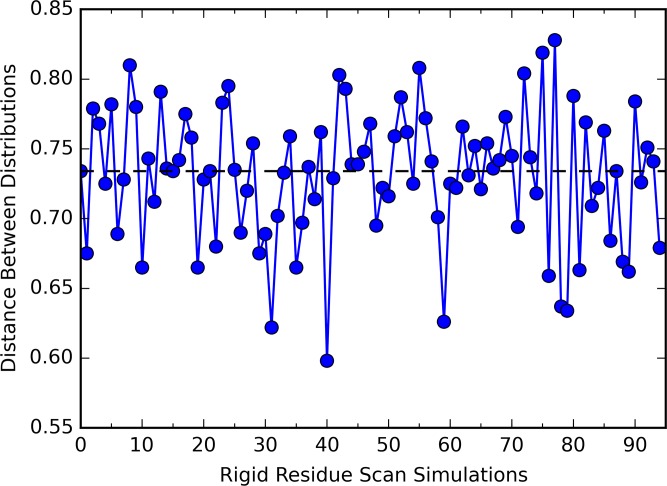
Distances between distributions of unbound and bound states from RRS simulations projecting onto the surfaces of two PC1 modes. Dashed line indicates the distance in the unperturbed simulations.

The simulations of unperturbed states are projected onto a 2D surface using two PC1 vectors from unperturbed states to illustrate the distribution of ensemble representing each state (Fig 9). Despite the close similarity between PDZ2 structures from unbound and bound states, the clear separation between two states on this 2D surface provides more insight into the allosteric difference between these states. The separation of two distributions on the 2D surface was represented by an average distance (0.734) between two attraction basins (Fig 9). All RRS simulations are also projected onto this 2D surface using the same two PC1 vectors to probe the impact of rigid residue perturbation on the distribution of ensemble on the same surface (S9 Table). For all the RRS simulations, the separation between unbound and bound states distributions resembles the unperturbed states, suggesting that the allosteric effect triggered by ligand binding event is robust upon rigid residue perturbation. However, the average distance between two distributions varies significantly among RRS simulations (Fig 10, S10 Table and sorted values in S11 Table), revealing the different contribution from each residue in the protein allostery. It is notable that the distribution distances of RRS simulations when residues R31, V40, or L78—all identified as key allosteric residues in the NMR study [72]—being held rigid are significantly shorter than the one of unperturbed states.

## Discussion

In the present study, we applied entropy analysis, quasi-harmonic analysis, and PCA on the RRS simulations of PDZ2 domain from PSD-95 protein to investigate the relationship between each individual residue and overall protein dynamics aiming to decipher protein allosteric mechanism within this framework.

### Residue Internal Dynamics and Overall Protein Dynamics

Individual amino acid residues, which are basic building blocks for protein structures, and therefore serve as the main target for many residue based protein allostery analysis methods,[47, 74–76] in which residue based interaction energy is the target for analysis. However, because protein allostery is mainly considered a dynamical process, it should be informative to investigate the internal dynamics of each individual residue and their impact on overall protein dynamics. Presumably, the internal degrees of freedom or dynamics of key allosteric residues should play unique roles in allostery with specific impact on overall protein dynamics. The RRS simulations combining with entropy analysis make it feasible to systematically evaluate the contribution from individual residue internal degrees of freedom to overall protein dynamics. Comparison between the unbound and bound states connects such contribution with protein allostery upon binding.

Rigid body constraint, which effectively removes the internal degrees of freedom in residue, should theoretically reduce the disorder of the protein as well as the protein entropy. On the contrary, rigid residue constraints lead to the increase of PDZ2 entropies in most RRS simulations. This counterintuitive observation indicates that the internal dynamics of each individual residue in a well-folded protein cooperatively contribute to the overall protein dynamics. In a recent simulation study of protein structures,[77] it was also reported that rigidifying some of protein degrees of freedom often cause more flexibility in other parts and lead to increasing protein entropy. The basic La Châtelier’s principle in chemical equilibrium was referred to govern the rigidity/flexibility equilibrium in protein structure.[77] Seemingly, our observation of increasing protein entropies in rigid residue simulations also agrees with the La Châtelier’s principle.

**Fig 11:**
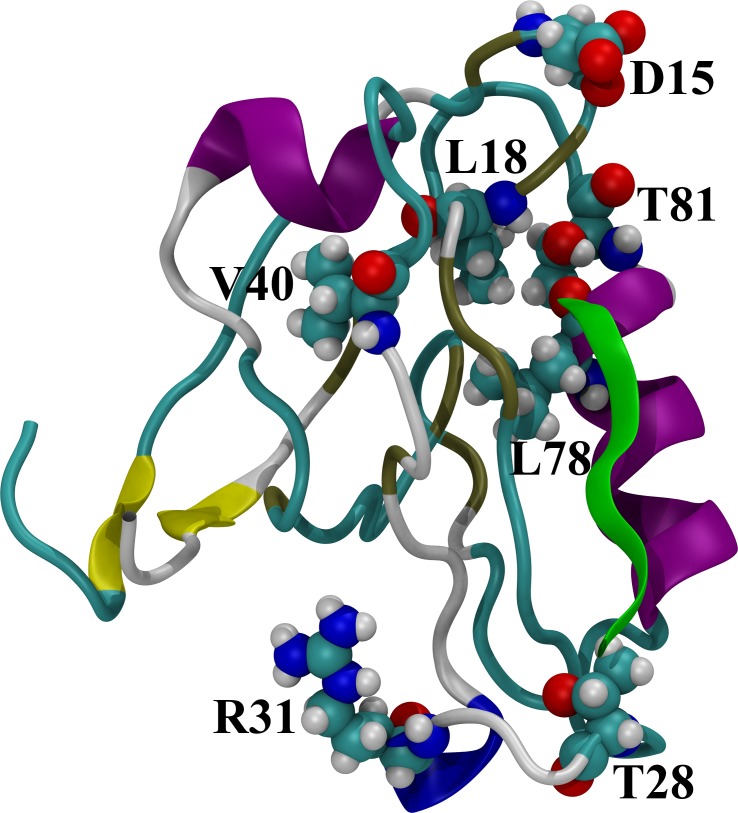
Key residues recognized based on protein entropic response to rigid body perturbation.

Without rigid body constraints, the binding with peptide leads to slight increase of PDZ2 entropy (0.016 kcal/(mol•K)). This is also in agreement with that the RMSD of PDZ2 in bound state is slightly higher than the one of unbound state (Fig 1). For only 11 residues among 94 PDZ2 residues, the PDZ2 entropy difference between unbound and bound states from RRS simulations is smaller than 0.016 kcal/(mol•K). Seven among these 11 residues D15, T28, V40, T81, R31, L78, L18 (Fig 11), were recognized as important for PDZ2 allostery upon binding by the NMR study.[72] All seven residues displayed significant dynamical parameter change upon binding. Although the direct relationship between the present study and NMR study of PDZ2 is not obvious, the overlap between two studies are unlikely to be random coincident. It should not be overlooked that the uncertainty of calculated configurational entropies undermines the reliability of predictions based on these calculations. Nevertheless, the current development is only a small step towards deeper understanding of protein allostery in terms of configurational entropy change. Improvement of configurational entropy calculations by including anharmonicity and higher order correlations will be applied to increase the reliability of the calculations.

The remaining residues were also identified by various computational studies as key allosteric residues. R79 was identified as one of “Hot Residues” for allostery in a study using ENM to probe PDZ2 allostery[58] as well as one of “nodes” to form an allostery communication network in another study using protein structure network model.[42] Both residues N14 and E90 were identified as part of an interacting cluster localized at the ligand binding pocket.[47] This strongly suggests that the RRS simulations could reveal the significance of internal dynamics of some key allosteric residues with regard to overall protein dynamics.

L18, as one of identified residues, when being held rigid, leads to the entropy increase of 0.045 kcal/(mol•K) for unbound state and 0.031 kcal/(mol•K) for bound state, which may be resulted from La Châtelier’s principle of protein rigidity/flexibility equilibrium.[77] However, this residue makes hydrophobic contact with the C-terminal valine of the binding peptide, which may counteract some of the entropy increasing effect. K13, another key residue for PDZ domain for interaction with binding peptide, forms multiple hydrogen bonds with Gly82, the backbone and side chain of Thr81. When K13 being held rigid, the entropy of PDZ2 increases 0.013 kcal/(mol•K) for unbound state and decreases 0.052 kcal/(mol•K) for bound state. These observations indicate that in RRS, strong interactions between residues being held rigid and ligand may help to counteract the general trend of increasing entropy in rigid body simulations.

### Response of Individual Residue to Perturbation

Another informative analysis presented in this study is the response from each individual residue to perturbations on proteins. By its definition, the mechanism of protein allostery is to be elucidated at overall protein structure level. However, this should not prevent any attempt to evaluate general response of individual residues to external or internal perturbations. Using the estimate of entropic contribution from individual residue in each simulation, the intrinsic difference of response to perturbation from each residue is revealed. Residue R31 is a clear case in the unbound state. Regardless which residue being held rigid, the entropic contribution from R31 is higher than the value in the unperturbed unbound state. Coincidentally, R31 has also been identified based on overall protein entropy change and NMR study[72] discussed in the previous section. On the contrary, this positive entropic response from R31 is inhibited upon binding with the peptide (Fig 5 and S3 and S4 Tables). Another six residues (T28, V61, L18, V26, V22, N27) also showed overwhelming positive response similar to R31, and were also recognized in the NMR study of PDZ2 allostery (Table 1).

The individual residue response pattern is significantly different in the bound state. Residues with consistent entropic response to rigid residue perturbations are very different from those in the unbound state. The observations that some residues display consistent response suggest that these residues are more sensitive to perturbations than other residues. The difference between unbound and bound states shows that the binding event inherently changes the sensitivity of each residue upon the perturbations, reflecting the very nature of protein allostery influenced by binding and perturbation events.

### Ensemble Distributions upon Coherent Allosteric Modes

Given the sheer number of modes generated from the quasi-harmonic analysis, one definitely suffers the risk of studying trivial and random patterns when focusing on only a few modes related to overall protein dynamics. However, the PCA analyses using multiple trajectories of unperturbed states clearly validate the physical significance of two PC1 modes from two states. The fact that two PC1 modes are virtually orthogonal to each other signifies the relevance between these two modes and protein allostery upon binding. The projection of RRS simulations onto these coherent allosteric modes support the entropy driven theory of protein allostery,[78, 79] which explain the protein allostery phenomenon as shifting of ensemble distribution upon perturbation instead of significant conformational change observed in either crystallographic[31] or NMR studies.[80] The various distances between two states projecting on the coherent allosteric modes demonstrate the shift of protein ensemble distribution upon rigid residue perturbation, revealing detailed information about individual residue with regard to overall allostery.

### Computational Cost of RRS Simulations

It is undeniable that the computational cost of the proposed method is exceptionally high compared to many other methods to elucidate protein allostery mechanisms. However, the current goal of our research is to develop an unbiased protocol to assess potential individual residue’s contribution towards overall protein dynamics and consequently allostery, with little or no *a priori* information about protein allosteric mechanisms. In the experiment, the most feasible way to probe the contribution of each individual residue to protein function is mutagenesis study. However, some knowledge of importance of residues is necessary for the mutagenesis study. Otherwise, all positions should be considered. In addition, it is somewhat arbitrary to choose what amino acids to which the wild type residues should be mutated. From the mutagenesis study, the perturbation added to protein contains two parts: removing the wild type residue and adding the mutated residue. These two parts are distinct but inseparable in the mutagenesis study. Without any *a priori* knowledge about relationship between protein sequence and allostery, one would mutate every residue to all other 19 natural amino acids to obtain the most comprehensive and unbiased evaluation of relationship between each residue and protein allostery. This is actually what has been done in an experimental study of allostery of another PDZ domain, in which total of 1577 mutants were generated for 83 out of 115 residues.[81] However the equivalent strategy could not be applied routinely to other proteins due to its obvious high cost. In addition to the exceedingly high cost of doing all possible mutations, the effects of removing the wild type residue and adding the mutated residue are still inseparable. One of the main advantages from the strategy presented in this study is that only the internal motion of each individual residue is removed, while the chemical content of the wild type residue is intact. This strategy provides a practical mean to investigate the intrinsic dynamical effect of each individual residue to overall protein dynamics. At this early stage, the RRS simulations were carried out for all residues for the sake of completion. Further improvement of the method is ongoing to significantly reduce the computational cost while keeping the confidence level of the results.

### Concluding Remarks

In this study, we further developed a recently proposed RRS method through combination of configurational entropy calculation and PCA to systematically evaluate the contribution of internal degrees of freedom of individual residue to overall protein dynamics and potential allostery upon ligand binding. Through the changes of the entropy from whole protein upon rigid residue perturbation, key residues were recognized as those when being held as rigid bodies, the protein entropy difference between unbound and bound states is smaller than the entropy difference from unperturbed simulations. These key residues have good agreement with a previous NMR study of the same protein bound to the same peptide.[72] Entropic response from individual residue upon perturbations was also evaluated. In the unbound state simulations, residues generally displaying increased entropic contribution upon rigid residue perturbation are in good agreement with the same NMR study.[72] The different patterns of individual residue response in the unbound and bound states suggest that the binding event inherently changes the sensitivity of each residue upon the perturbations. PCA of unperturbed states of PDZ2 revealed two quasi-harmonic coherent allosteric modes, which are robust upon analysis of multiple trajectories of each state. The projection of RRS simulations onto coherent allosteric modes reveals the intrinsic shifting of ensemble distributions upon rigid residue perturbations, and supports the population-shift point of view about protein allostery. Overall, the combination of entropy calculation and PCA with the RRS method provides a systematic approach to estimate the individual residue contribution to protein dynamics as well as allostery. Further development is actively under development to reduce the computational cost with deeper understanding of the protein allostery.

## Methods

### MD Simulations

The assessment of the role of individual residues in overall protein dynamics is carried out using rigid residue scan, a systematic simulation method developed in our group.[68] In the RRS method, rigid body constraints are applied to each residue in the target protein in separate simulations (referred to as perturbed simulations). Thus, there are as many rigid body MD simulations (perturbed simulations) as there are residues comprising the target protein.

The second PDZ domain (PDZ2) from the human tyrosine phosphatase 1E (hPTP1E) is used as a test protein in this study to further develop the RRS method. The allosteric mechanisms of PDZ2 have been the subject of a number of studies with various residues identified as key allosteric residues.[42, 47, 61] Initial crystal structures for the unbound and bound states with RA-GEF2 peptide (EQVSAV) were obtained from the Protein Data Bank (PDB) with IDs 3LNX and 3LNY, respectively.[82] PDZ2 structures in both 3LNX and 3LNY contain 94 residues. For the residues with multiple copies in the PDB files, the first coordinate set was used to prepare the simulation systems.

The structures from the PDB were processed with hydrogen atoms added and solvated in water (TIP3P)[83] with charge balancing ions of sodium and chlorine added. Additional ions were included to adjust the ionic strength in simulation cells to about 0.02 M. The system was then subjected to energy minimization with 200 steps of steepest descent and 9491 steps of adopted basis Newton-Raphson minimization, which yielded a total gradient of less than 0.001 kcal/(mol•Å). This was followed by an equilibration step that raised the temperature of the systems from 100 K to 300 K over 12 picoseconds (ps). Then the systems were equilibrated via 10 nanosecond (ns) isothermal-isobaric ensemble (NPT) MD simulations at 300 K and 1 atm. The frame from the simulation trajectory with dimensions closest to the average dimension for the entire trajectory was selected. This set of coordinates and its corresponding velocities were used as the initial conditions for 34 ns canonical ensemble (NVT) Langevin MD simulations also at 300 K. The first 4 ns of each NVT simulation was treated as equilibrium, and therefore not included in the reported analysis. The NVT simulations consisted of normal MD simulations without rigid residue constraint for the unbound and bound PDZ2 (referred as unperturbed simulations) and the rigid residue scan over all 94 residues in PDZ2. There are total of 190 simulations, including 188 rigid residue simulations and two unperturbed simulations of unbound and bound states of PDZ2. Considering 30 ns of each NVT simulation for analysis, this work comprises 5,700 ns of simulation time. A 2 femtosecond (fs) simulation time step was used in all simulations. To estimate the error bar of the entropy calculations and validate coherent allosteric modes, additional 180 ns simulations were carried out for the unperturbed states and rigid residue simulations corresponding to seven residues (15, 18, 22, 28, 40, 61, and 81). Each set of 180 ns simulations were carried out with three independent 60 ns trajectories for better sampling and shorter computing times. All simulations used cubic periodic boundary conditions, and electrostatic interactions were modeled using the particle mesh Ewald method.[84] All simulations were carried out using CHARMM version 38b1 and version 27 of the CHARMM force field.[85]

### Analysis of MD Trajectories

#### RMSD

The RMSD was used to measure the variability of a set of Cartesian coordinates over the course of a MD trajectory relative to a reference set. Specifically, the coordinates of each atom comprising the protein or a subset are compared with that of a reference structure. The RMSD for a given simulation is defined as

$$R\;=\sqrt{\frac{\sum_{i=1}^{N}{\left({\text{r}}_{i}^{0}-{\text{Ur}}_{i}\right)}^{2}}{N}},$$

where *N* is the number of atoms, r_i_^0^ is the Cartesian coordinate vector for atom *I*, and U is the best-fit alignment transformation matrix between a given structure and its reference structure.

#### Cross-correlation (normalized covariance) matrix

The cross-correlation matrix is a measure of the correlated movement of a set of atoms. Each matrix element is defined as

$${\text{C}}_{\text{ij}}=\frac{{\text{c}}_{\text{ij}}}{{\text{c}}_{\text{ii}}^{1/2}{\text{c}}_{\text{jj}}^{1/2}}=\frac{\langle {\text{r}}_{\text{i}}{\text{r}}_{\text{j}}\rangle -\langle {\text{r}}_{\text{i}}\rangle \langle {\text{r}}_{\text{j}}\rangle }{{\left[\left(\langle {\text{r}}_{\text{i}}^{2}\rangle -{\langle {\text{r}}_{\text{i}}\rangle }^{2}\right)\left(\langle {\text{r}}_{\text{j}}^{2}\rangle -{\langle {\text{r}}_{\text{j}}\rangle }^{2}\right)\right]}^{1/2}},$$

where C_ij_ is the measure of correlated movement between atoms i and j, c_ij_, c_ii_, and c_jj_ are the covariance matrix elements, and r_i_ and r_j_ are Cartesian coordinate vectors from the least-square fitted structures, therefore with translation and rotation projected out. Matrix elements are between -1 and 1 with negative values indicating negative correlation and positive values indicating positive correlation between the motions of atoms i and j. Correlation is defined as related movement along the line between two points. Correlated movement along orthogonal paths yields a cross-correlation matrix element of zero.[86] After discarding the first 4 ns NVT simulation as equilibrium for each simulation, total of 15,000 frames were extracted from the remaining 30ns simulation with 2ps interval, and processed to generate cross-correlation matrix.

#### Entropy analysis

All-atom quasi-harmonic analysis was employed to analyze MD trajectories to probe protein dynamics using vibrational normal modes on an effective quasi-harmonic potential.[87] The element of force constant matrix **F** on the effective quasi-harmonic potential for normal modes calculation is given by [70]

$${\text{F}}_{ij}\;=\;k_{B}T{\left[{\text{C}}^{-1}\right]}_{ij},$$

where *k*_*B*_ is the Boltzmann constant, *T* is the temperature, and [C^-1^] is the inverse of covariance matrix C. Therefore, the normal modes and corresponding frequency ω of the molecule on the effective quasi-harmonic potential can be calculated through the solution of secular equation

$$\text{det}(F-\omega^{2}M)=0,$$

where **M** is the mass matrix of protein. The configurational entropy of protein, *S*_*config*_ could be estimated from the 3*n*−6 nonzero quasi-harmonic frequencies through [71]

$$S_{config}\;=\;k_{B}\sum_{i}^{3n-6}\frac{\hbar \omega_{i}/kT}{e^{\hbar \omega_{i}/kT}-1}-\text{ln}\left(1-e^{-\hbar \omega_{i}/kT}\right).$$

It should be noted that the *S*_*config*_ is an approximation of the target value using the exact equation within harmonic limit. The all-atom covariance matrices were employed for entropy calculations. Because the protein structures from each simulation are superimposed to the first frame of the simulation before the calculation of covariance matrix, the overall translation and rotation motion was projected out for the entropy calculation.

To estimate entropy contribution from each residue, all-atom covariance matrix elements corresponding to correlation between atoms within the same residue (including side chain and backbone) are selected to form a sub covariance matrix, which is processed in the same way described above.[85, 88, 89] By constructing a sub covariance matrix for each residue from all-atom covariance matrix of whole protein, no alignment of the different conformations of the subsystem was performed. It should also be noted that the entropy corresponding to the correlation between the target residue and the rest of the system is not included in the individual residue entropy.

#### Principal component analysis (PCA)

For each simulation, PCA was performed by projecting each of the extracted 15,000 frames from 30 ns trajectory onto the normal modes generated by quasi-harmonic analysis. Both translation and rotation components were projected out for each frame. The residues being held rigid were included in the above analyses just as any other residues. All the analyses described in this section were carried out using CHARMM version 38b1.[85]

## Supporting Information

S1 Fig Autocorrelation functions of unperturbed and seven rigid residue scan simulations. Relaxation time around 20 ps was displayed in all simulations.
(TIF)

S2 Fig Heat maps of normalized individual residue entropy contribution under rigid residue perturbation for unbound (left) and bound (right) states. The entropy contribution from each residue in unperturbed simulations (with index as 0 in both plots) is set as reference.
(TIF)

S3 Fig Average of normalized entropic response from each residue in all rigid residue scan simulations. (TIF)

S4 Fig Distribution of density of states for all rigid residue scan simulations. (TIF)

S1 Table RMSD plots of PDZ2 from rigid residue scan for both unbound and bound states. (PDF)

S2 Table Heat maps, histograms of Cα carbons cross-correlation matrices for all residues in PDZ2 from unperturbed and rigid residue scan simulations. (PDF)

S3 Table Relative entropies (ΔS) and differences (ΔΔS) of PDZ2 between unbound and bound states. (PDF)

S4 Table Relative entropies (ΔS) and differences (ΔΔS) of PDZ2 between unbound and bound states sorted with ascending order. (PDF)

S5 Table Average entropic response of individual residues upon rigid body perturbations. (PDF)

S6 Table Average entropic response of individual residues upon rigid body perturbations sorted with descending order. (PDF)

S7 Table Dot products of five lowest frequency quasi-harmonic modes (PC1-PC5) from seven sets of 30 ns trajectories with the PC1 to PC5 from whole 210 ns trajectory. (PDF)

S8 Table Dot products among PC1 modes from seven 30 ns trajectories. (PDF)

S9 Table Projections of simulations onto 2D-surface using two PC1 modes from unperturbed unbound and bound states. (PDF)

S10 Table Average distance between distributions of unbound and bound states projected onto 2D-surface using two PC1 modes. (PDF)

S11 Table Average distance between distributions of unbound and bound states projected onto 2D-surface using two PC1 modes sorted with ascending order. (PDF)
