# Supplementary figures and images for: Rigid Residue Scan Simulations Systematically Reveal Residue Entropic Roles in Protein Allostery
Source: PLoS Comput Biol. 2016 Apr 26;12(4):e1004893. doi: 10.1371/journal.pcbi.1004893 (PMC4846164; doi:10.1371/journal.pcbi.1004893)

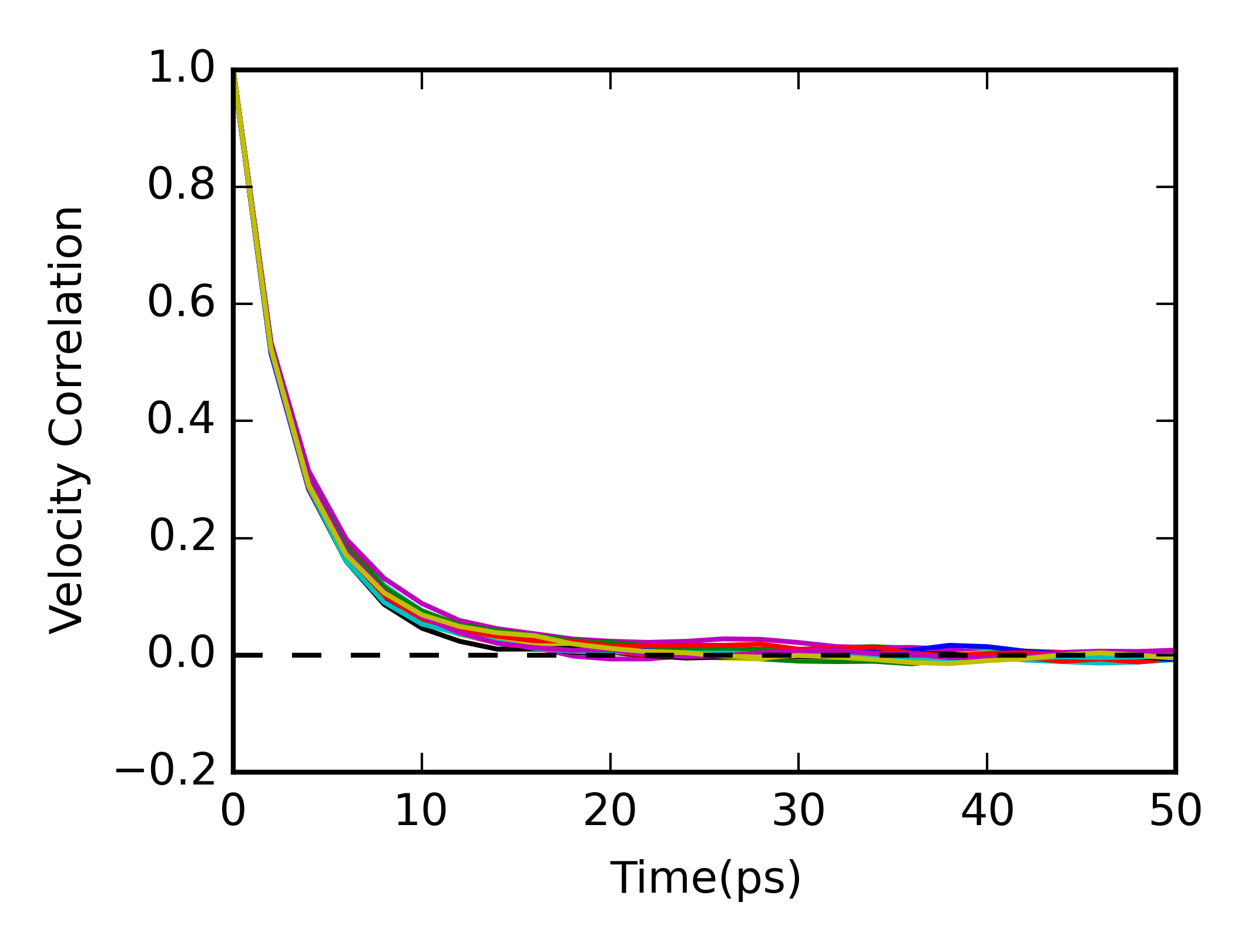

Supplement: S1 Fig — Relaxation time around 20 ps was displayed in all simulations. (TIF) [file pcbi.1004893.s001.tif]

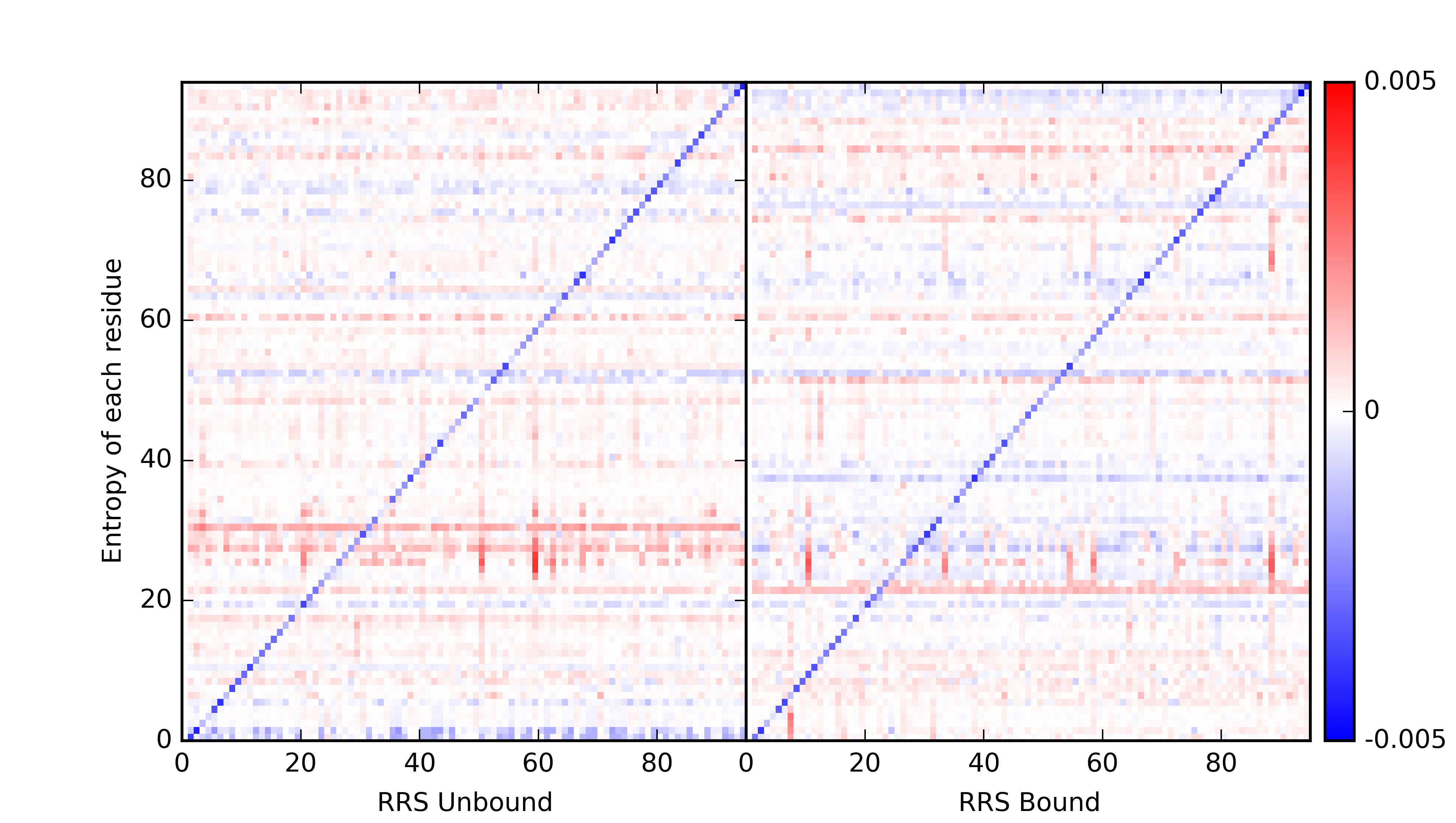

Supplement: S2 Fig — The entropy contribution from each residue in unperturbed simulations (with index as 0 in both plots) is set as reference. (TIF) [file pcbi.1004893.s002.tif]

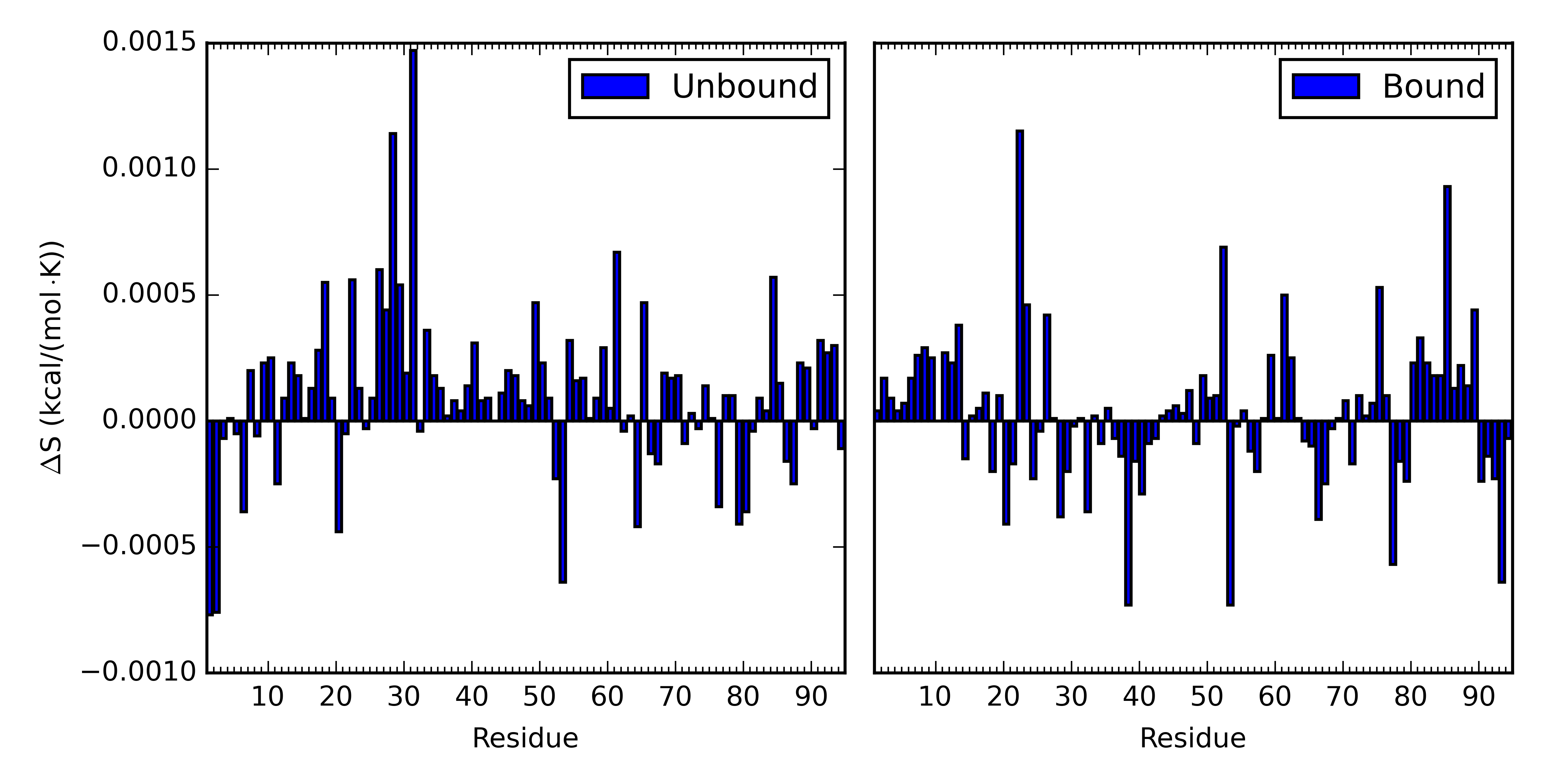

Supplement: S3 Fig — (TIF) [file pcbi.1004893.s003.tif]

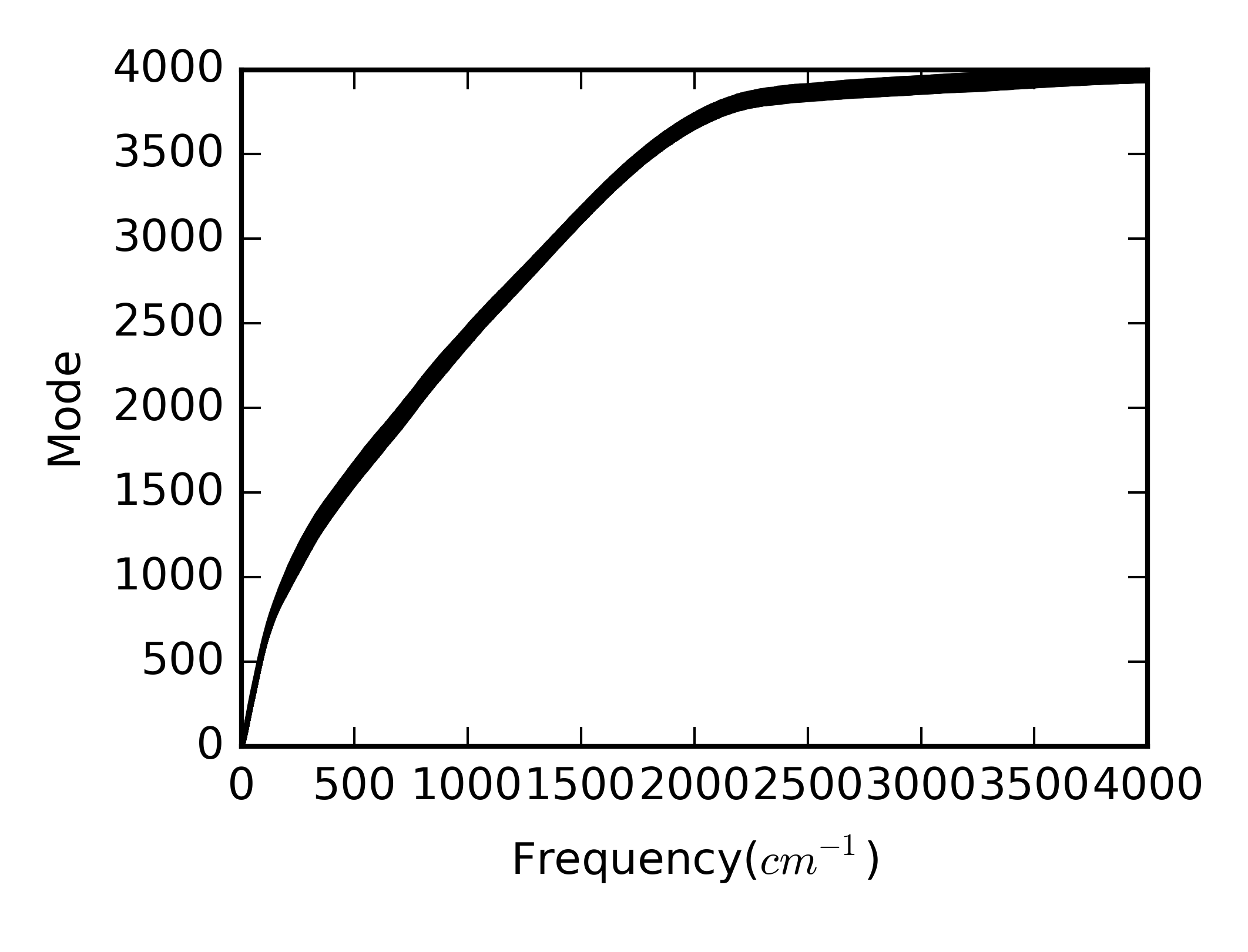

Supplement: S4 Fig — (TIF) [file pcbi.1004893.s004.tif]
