# Supplementary material for: Rigid Residue Scan Simulations Systematically Reveal Residue Entropic Roles in Protein Allostery
Source: PLoS Comput Biol. 2016 Apr 26;12(4):e1004893. doi: 10.1371/journal.pcbi.1004893 (PMC4846164; doi:10.1371/journal.pcbi.1004893)
Supplement: S1 Table — (PDF) [file pcbi.1004893.s005.pdf]

Table S1: RMSD plots of PDZ2 from rigid residue scan for both unbound and bound states. Each residue number represents a simulation in which that particular residue is subjected to rigid body constraints.

<table>
<tr><th>Residue</th><th>Unbound</th><th>Bound</th><th>Residue</th><th>Unbound</th><th>Bound</th></tr>
<tr><td>None</td><td colspan="2">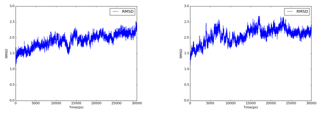</td><td></td><td colspan="2"></td></tr>
<tr><td>1</td><td colspan="2">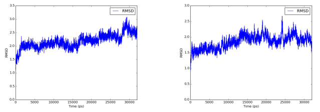</td><td>2</td><td colspan="2">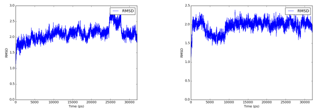</td></tr>
<tr><td>3</td><td colspan="2">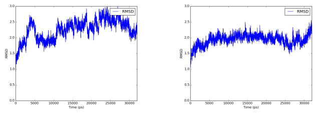</td><td>4</td><td colspan="2">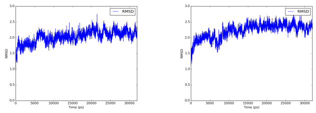</td></tr>
<tr><td>5</td><td colspan="2">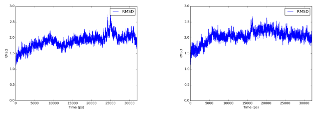</td><td>6</td><td colspan="2">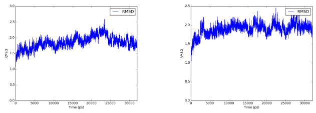</td></tr>
<tr><td>7</td><td colspan="2">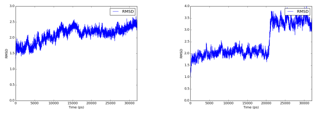</td><td>8</td><td colspan="2">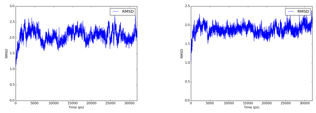</td></tr>
<tr><td>9</td><td colspan="2">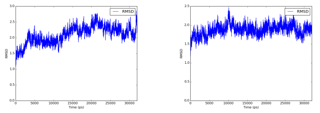</td><td>10</td><td colspan="2">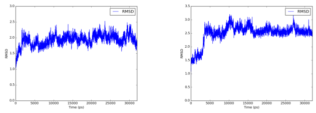</td></tr>
<tr><td>11</td><td colspan="2">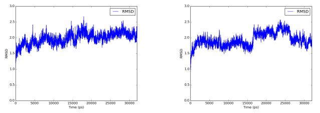</td><td>12</td><td colspan="2">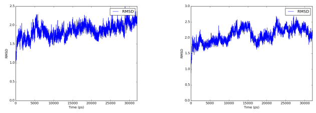</td></tr>
<tr><td>13</td><td colspan="2">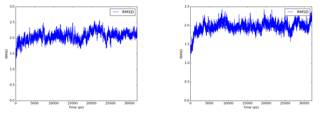</td><td>14</td><td colspan="2">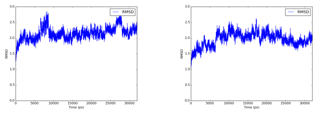</td></tr>
<tr><td>15</td><td colspan="2">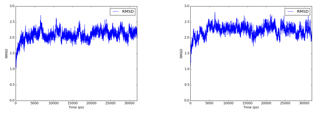</td><td>16</td><td colspan="2">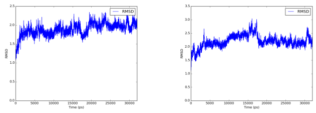</td></tr>
<tr><td>17</td><td colspan="2">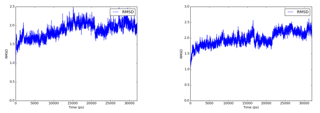</td><td>18</td><td colspan="2">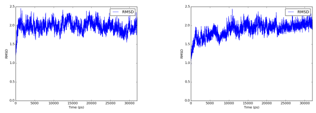</td></tr>
<tr><td>19</td><td colspan="2">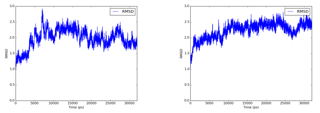</td><td>20</td><td colspan="2">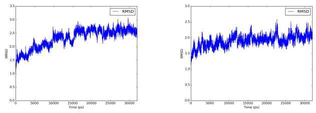</td></tr>
<tr><td>21</td><td colspan="2">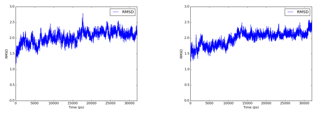</td><td>22</td><td colspan="2">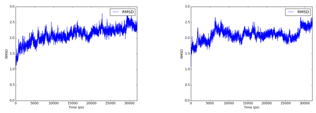</td></tr>
</table>

Table S1: RMSD plots of PDZ2 from rigid residue scan for both unbound and bound states. Each residue number represents a simulation in which that particular residue is subjected to rigid body constraints.

Residue	Unbound	Bound	Residue	Unbound	Bound
23	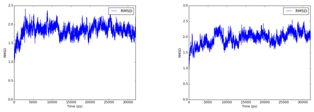		24	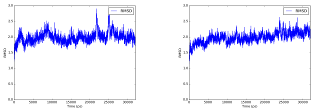	
25	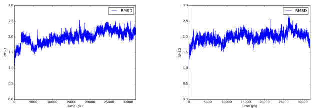		26	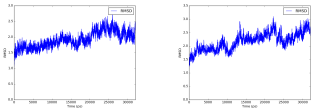	
27	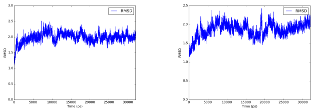		28	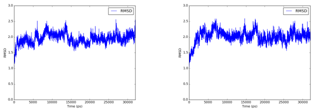	
29	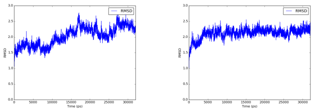		30	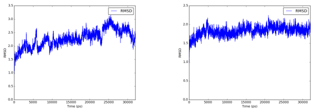	
31	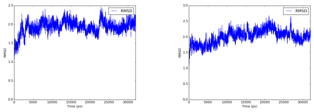		32	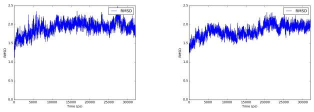	
33	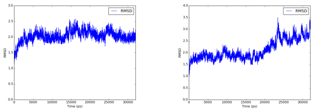		34	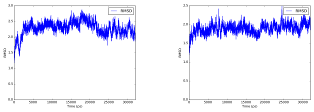	
35	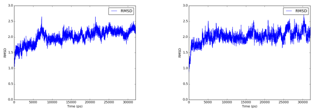		36	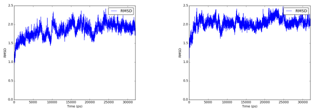	
37	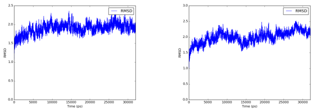		38	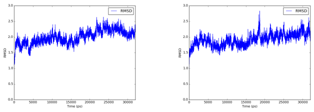	
39	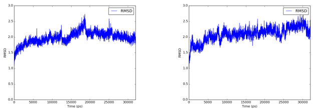		40	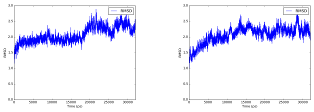	
41	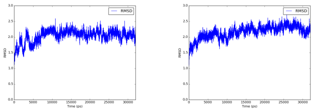		42	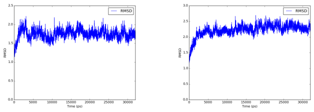	
43	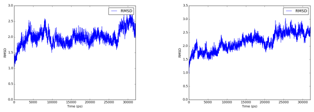		44	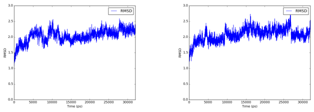	
45	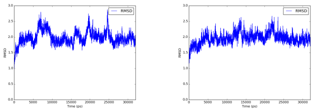		46	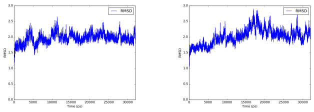	

Table S1: RMSD plots of PDZ2 from rigid residue scan for both unbound and bound states. Each residue number represents a simulation in which that particular residue is subjected to rigid body constraints.

Residue	Unbound	Bound	Residue	Unbound	Bound
47	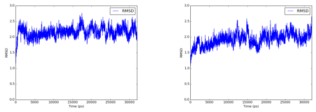		48	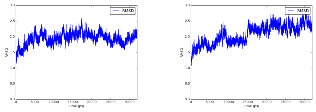	
49	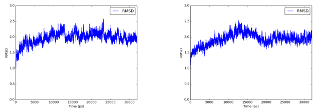		50	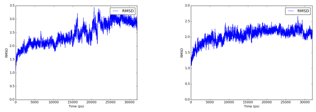	
51	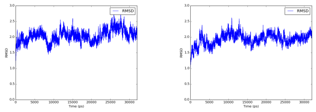		52	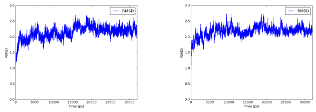	
53	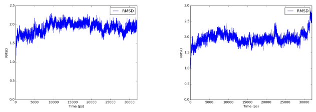		54	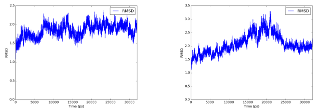	
55	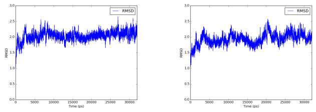		56	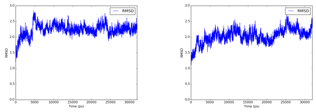	
57	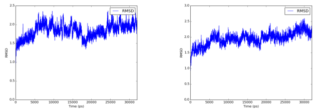		58	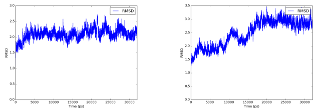	
59	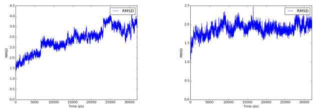		60	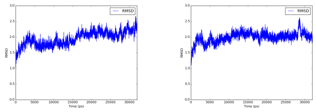	
61	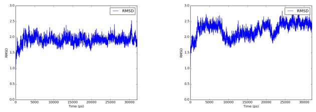		62	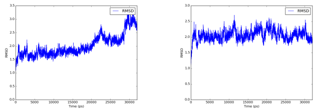	
63	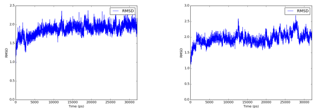		64	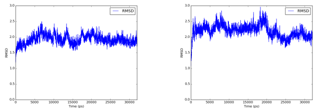	
65	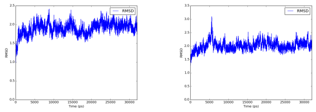		66	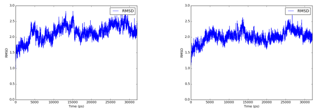	
67	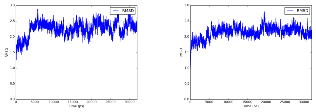		68	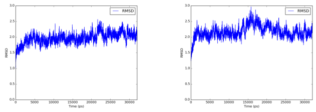	
69	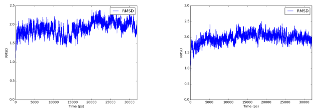		70	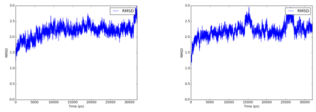	

Table S1: RMSD plots of PDZ2 from rigid residue scan for both unbound and bound states. Each residue number represents a simulation in which that particular residue is subjected to rigid body constraints.

Residue	Unbound	Bound	Residue	Unbound	Bound
71	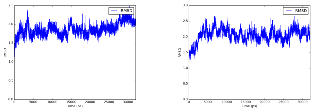		72	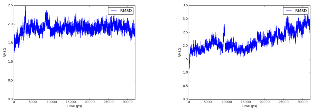	
73	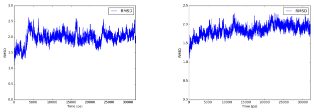		74	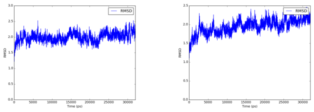	
75	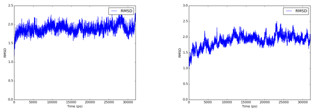		76	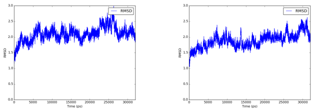	
77	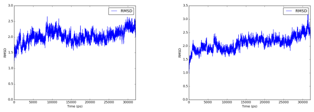		78	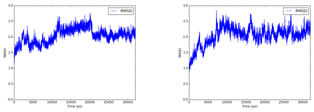	
79	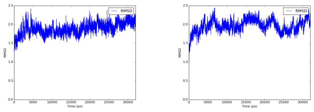		80	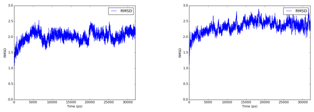	
81	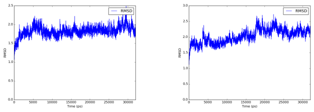		82	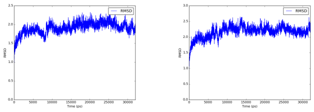	
83	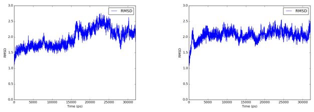		84	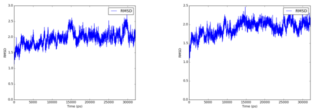	
85	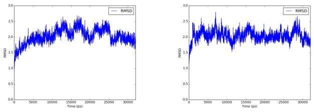		86	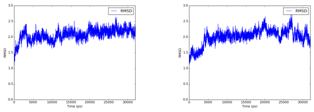	
87	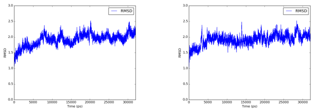		88	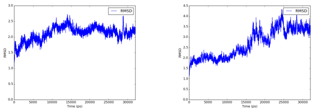	
89	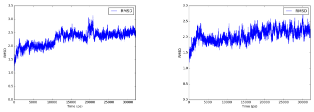		90	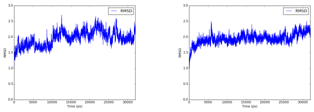	
91	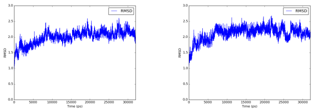		92	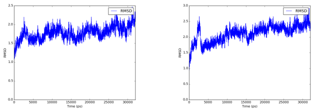	
93	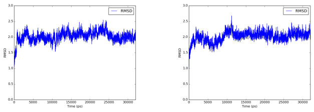		94	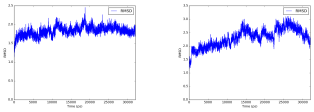	
