# Supplementary material for: Rigid Residue Scan Simulations Systematically Reveal Residue Entropic Roles in Protein Allostery
Source: PLoS Comput Biol. 2016 Apr 26;12(4):e1004893. doi: 10.1371/journal.pcbi.1004893 (PMC4846164; doi:10.1371/journal.pcbi.1004893)
Supplement: S2 Table — (PDF) [file pcbi.1004893.s006.pdf]

Table S2: Heat maps, histograms of Cα cross-correlation matrices for all residues in PDZ2 from rigid residue scan for both unbound and bound states. Each residue number represents a simulation in which that particular residue is subjected to rigid body constraints.

	Heat Map[a]		Histogram[b]	
Residue	Unbound	Bound	Unbound	Bound
None	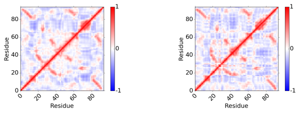		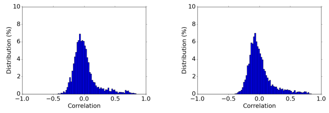	
1	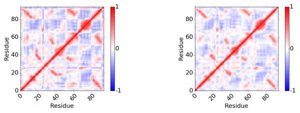		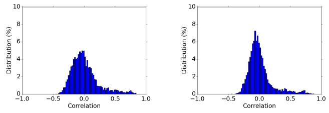	
2	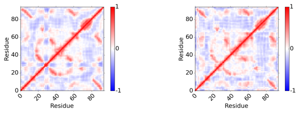		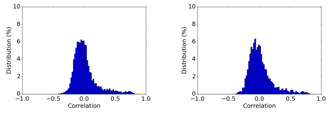	
3	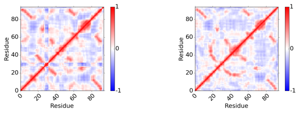		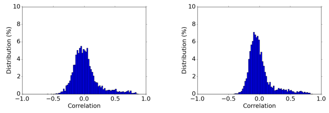	
4	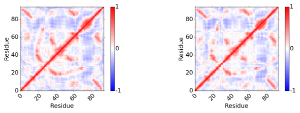		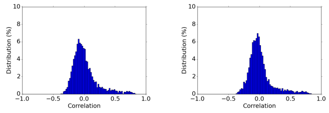	
5	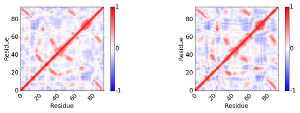		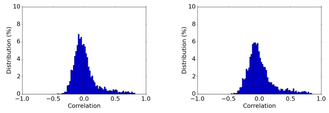	
6	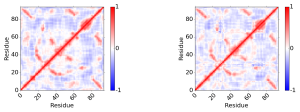		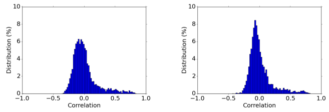	
7	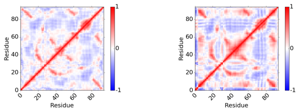		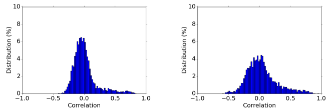	
8	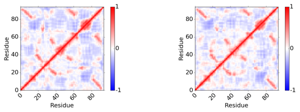		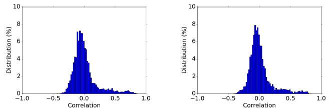	
9	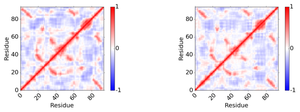		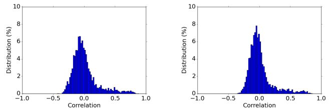	
10	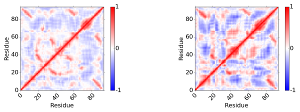		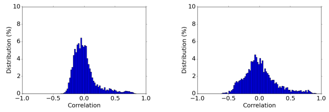	

[a] In these heat maps, red means positive correlation, blue means negative correlation, white means no correlation.
[b] In the histogram plots, normalized distribution is plotted using 0.2 as bin width.

Table S2: Heat maps, histograms of Cα cross-correlation matrices for all residues in PDZ2 from rigid residue scan for both unbound and bound states. Each residue number represents a simulation in which that particular residue is subjected to rigid body constraints.

	Heat Map[a]		Histogram[b]	
Residue	Unbound	Bound	Unbound	Bound
11	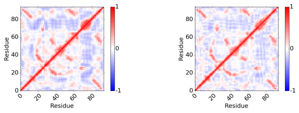		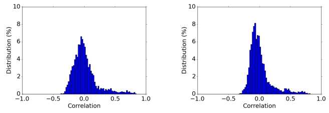	
12	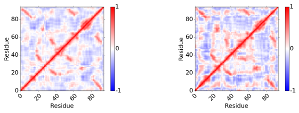		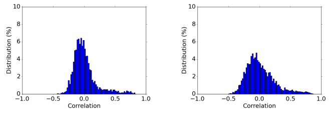	
13	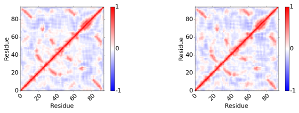		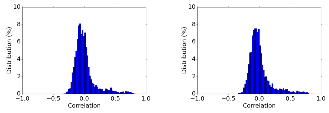	
14	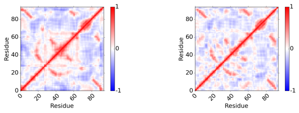		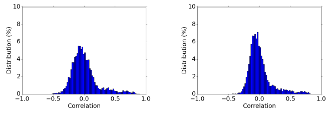	
15	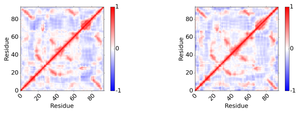		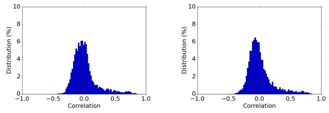	
16	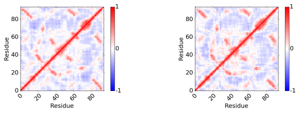		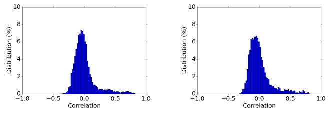	
17	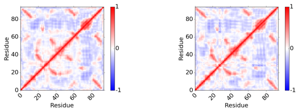		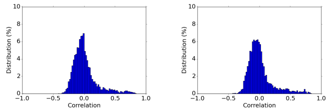	
18	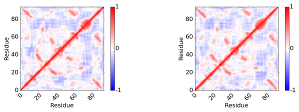		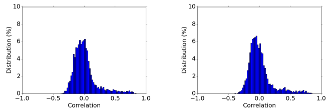	
19	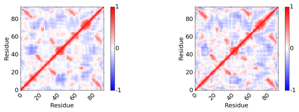		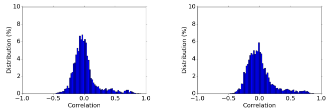	
20	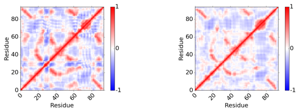		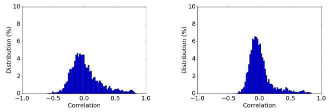	
21	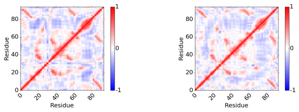		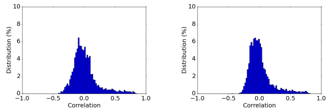	

[a] In these heat maps, red means positive correlation, blue means negative correlation, white means no correlation.
[b] In the histogram plots, normalized distribution is plotted using 0.2 as bin width.

Table S2: Heat maps, histograms of Cα cross-correlation matrices for all residues in PDZ2 from rigid residue scan for both unbound and bound states. Each residue number represents a simulation in which that particular residue is subjected to rigid body constraints.

	Heat Map[a]		Histogram[b]	
Residue	Unbound	Bound	Unbound	Bound
22	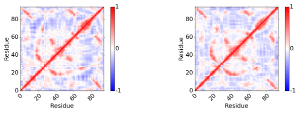		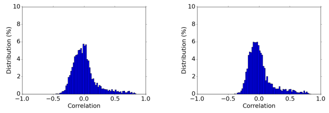	
23	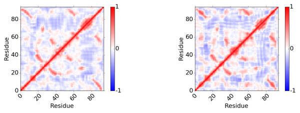		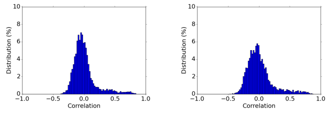	
24	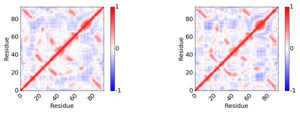		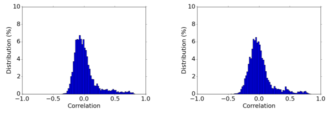	
25	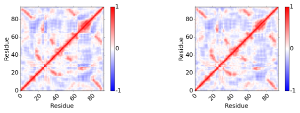		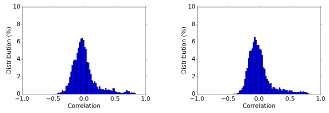	
26	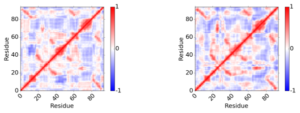		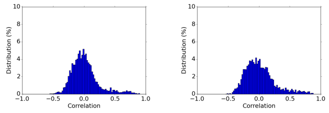	
27	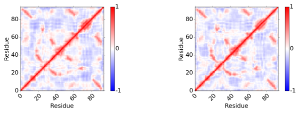		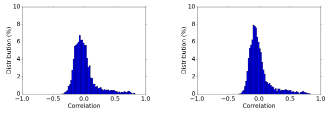	
28	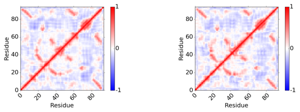		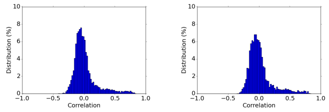	
29	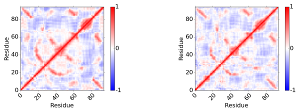		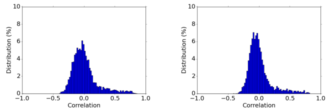	
30	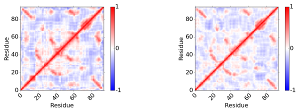		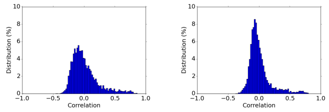	
31	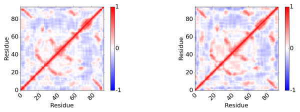		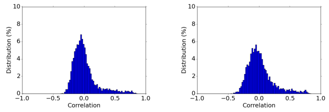	
32	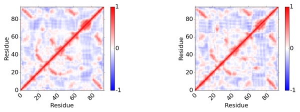		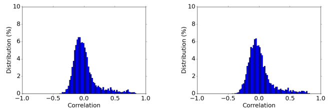	

[a] In these heat maps, red means positive correlation, blue means negative correlation, white means no correlation.

[b] In the histogram plots, normalized distribution is plotted using 0.2 as bin width.

Table S2: Heat maps, histograms of Cα cross-correlation matrices for all residues in PDZ2 from rigid residue scan for both unbound and bound states. Each residue number represents a simulation in which that particular residue is subjected to rigid body constraints.

	Heat Map[a]	Histogram[b]
Residue	Unbound / Bound	Unbound / Bound
33	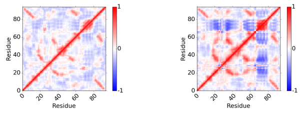	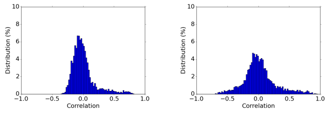
34	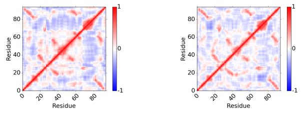	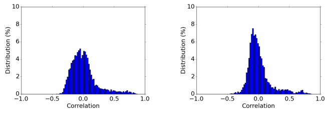
35	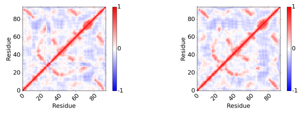	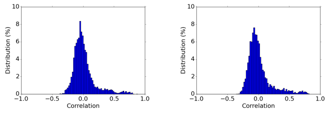
36	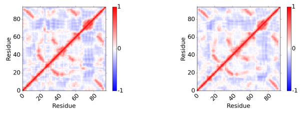	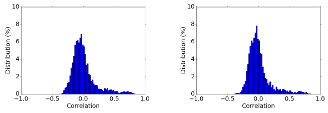
37	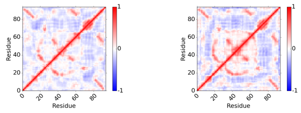	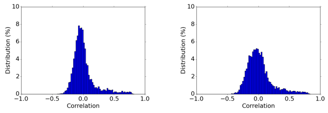
38	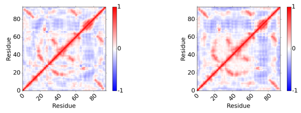	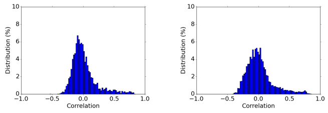
39	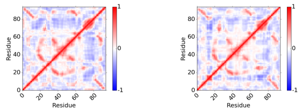	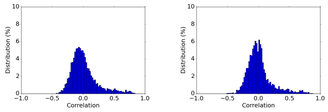
40	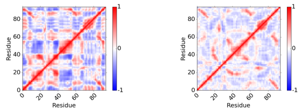	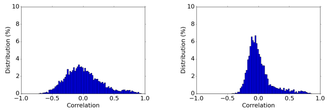
41	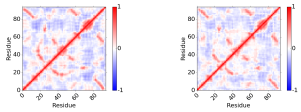	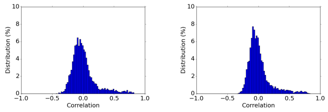
42	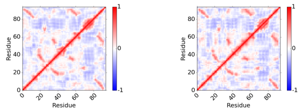	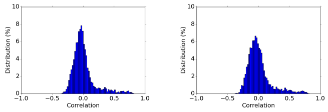
43	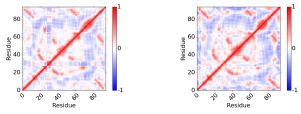	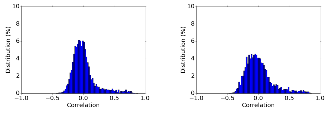

[a] In these heat maps, red means positive correlation, blue means negative correlation, white means no correlation.
[b] In the histogram plots, normalized distribution is plotted using 0.2 as bin width.

Table S2: Heat maps, histograms of Cα cross-correlation matrices for all residues in PDZ2 from rigid residue scan for both unbound and bound states. Each residue number represents a simulation in which that particular residue is subjected to rigid body constraints.

	Heat Map[a]		Histogram[b]	
Residue	Unbound	Bound	Unbound	Bound
44	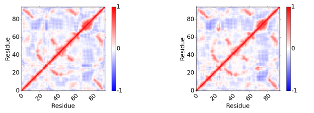		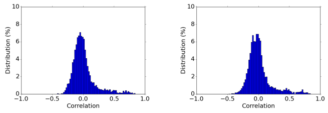	
45	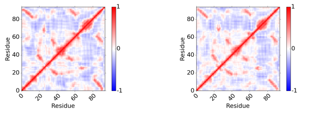		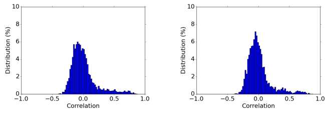	
46	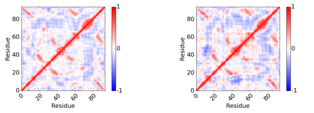		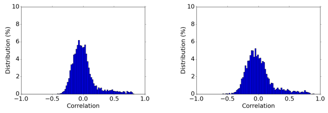	
47	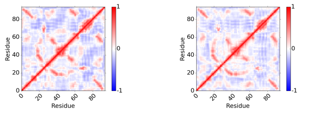		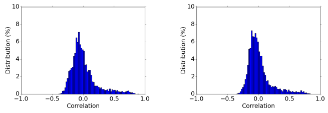	
48	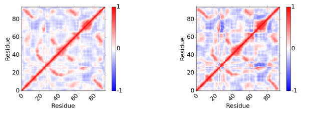		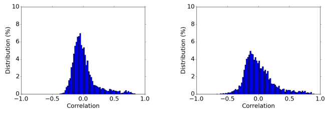	
49	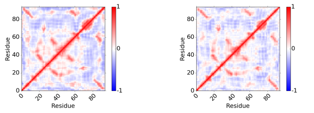		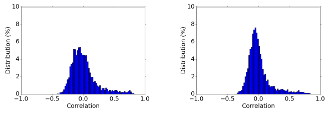	
50	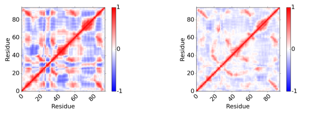		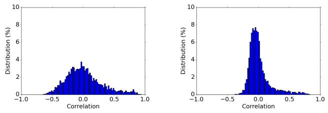	
51	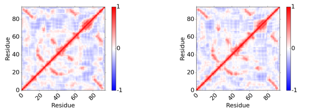		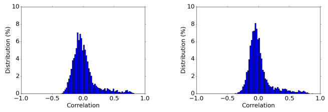	
52	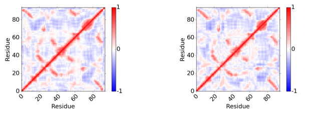		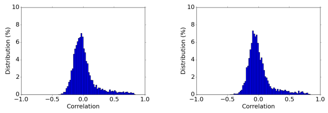	
53	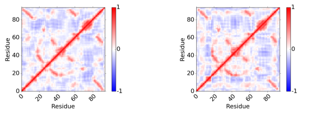		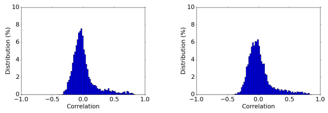	
54	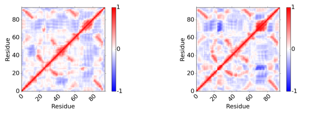		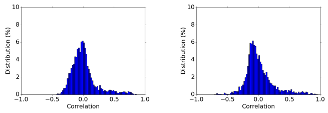	

[a] In these heat maps, red means positive correlation, blue means negative correlation, white means no correlation.
[b] In the histogram plots, normalized distribution is plotted using 0.2 as bin width.

Table S2: Heat maps, histograms of Cα cross-correlation matrices for all residues in PDZ2 from rigid residue scan for both unbound and bound states. Each residue number represents a simulation in which that particular residue is subjected to rigid body constraints.

	Heat Map[a]		Histogram[b]	
Residue	Unbound	Bound	Unbound	Bound
55	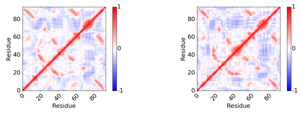		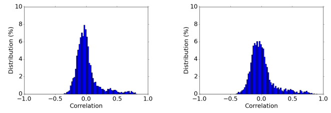	
56	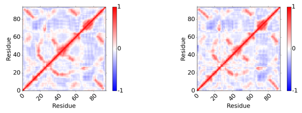		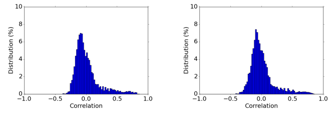	
57	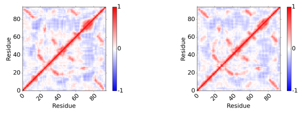		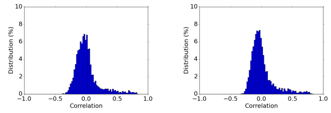	
58	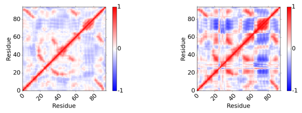		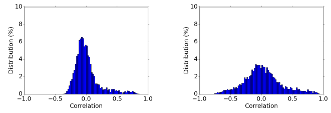	
59	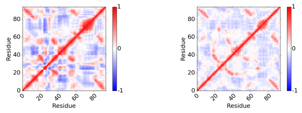		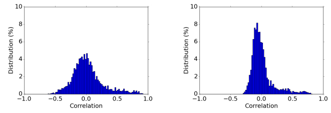	
60	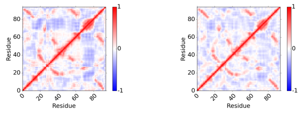		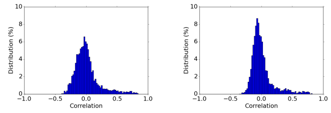	
61	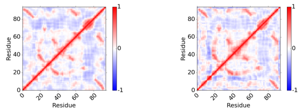		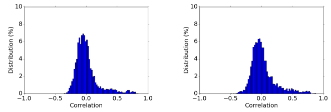	
62	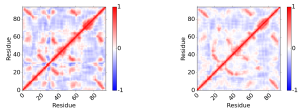		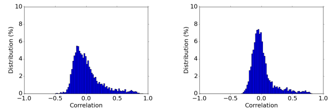	
63	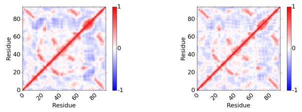		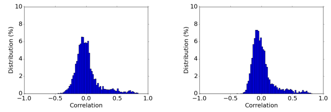	
64	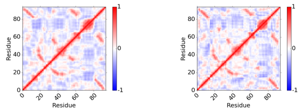		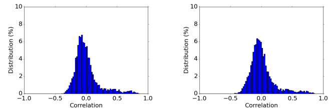	
65	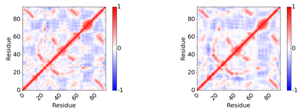		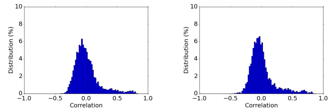	

[a] In these heat maps, red means positive correlation, blue means negative correlation, white means no correlation.
[b] In the histogram plots, normalized distribution is plotted using 0.2 as bin width.

Table S2: Heat maps, histograms of Cα cross-correlation matrices for all residues in PDZ2 from rigid residue scan for both unbound and bound states. Each residue number represents a simulation in which that particular residue is subjected to rigid body constraints.

Residue	Heat Map[a] (Unbound, Bound)	Histogram[b] (Unbound, Bound)
66	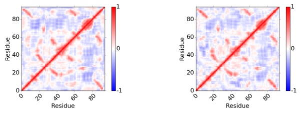	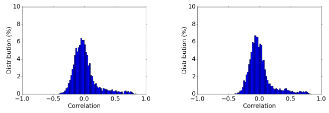
67	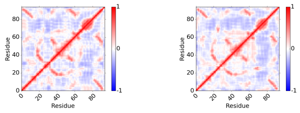	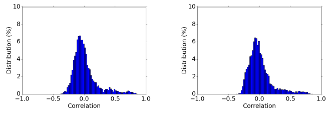
68	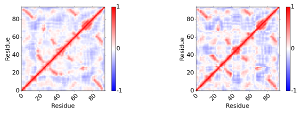	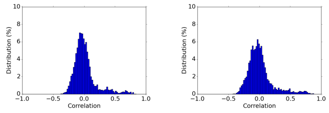
69	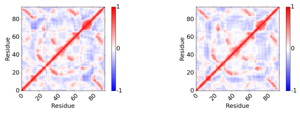	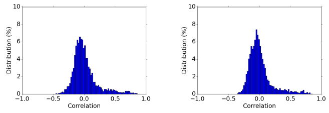
70	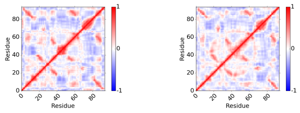	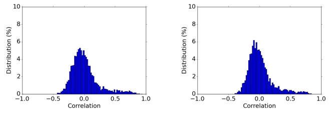
71	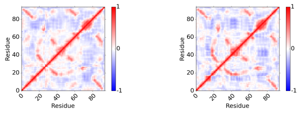	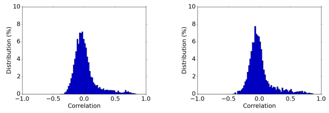
72	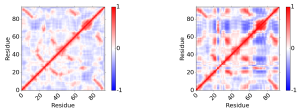	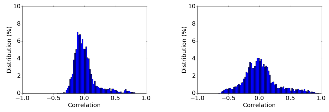
73	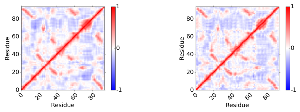	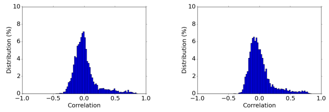
74	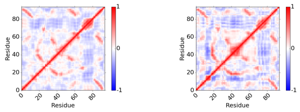	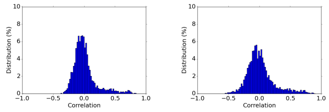
75	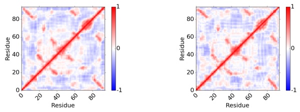	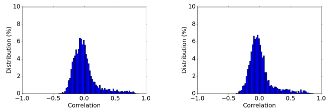
76	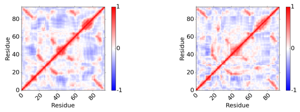	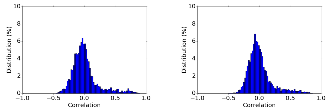

[a] In these heat maps, red means positive correlation, blue means negative correlation, white means no correlation.
[b] In the histogram plots, normalized distribution is plotted using 0.2 as bin width.

Table S2: Heat maps, histograms of Cα cross-correlation matrices for all residues in PDZ2 from rigid residue scan for both unbound and bound states. Each residue number represents a simulation in which that particular residue is subjected to rigid body constraints.

	Heat Map[a]		Histogram[b]	
Residue	Unbound	Bound	Unbound	Bound
77	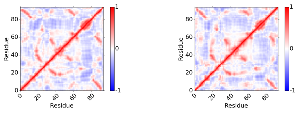		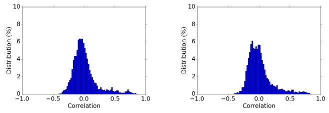	
78	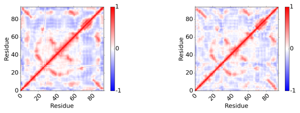		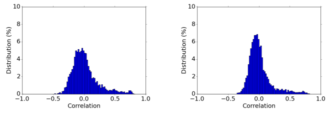	
79	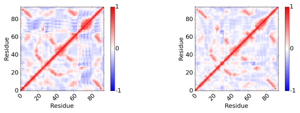		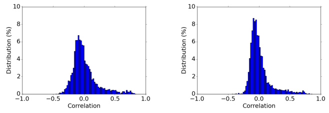	
80	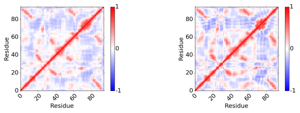		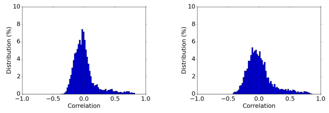	
81	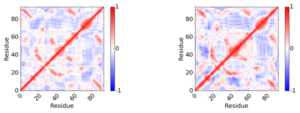		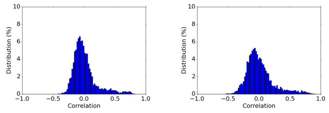	
82	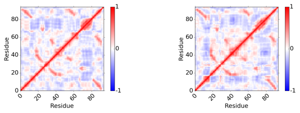		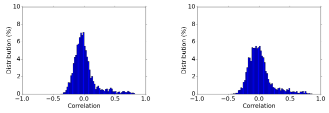	
83	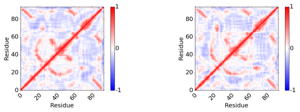		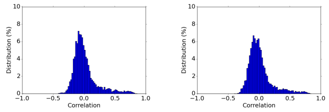	
84	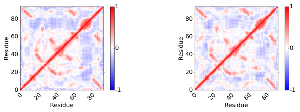		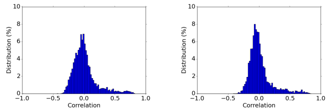	
85	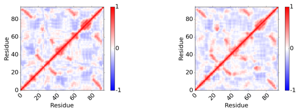		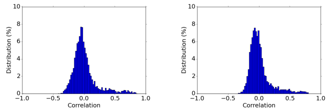	
86	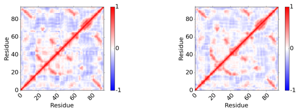		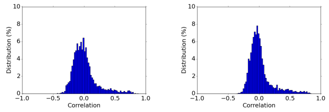	
87	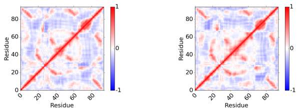		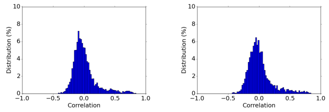	

[a] In these heat maps, red means positive correlation, blue means negative correlation, white means no correlation.
[b] In the histogram plots, normalized distribution is plotted using 0.2 as bin width.

Table S2: Heat maps, histograms of Cα cross-correlation matrices for all residues in PDZ2 from rigid residue scan for both unbound and bound states. Each residue number represents a simulation in which that particular residue is subjected to rigid body constraints.

	Heat Map[a]		Histogram[b]	
Residue	Unbound	Bound	Unbound	Bound
88	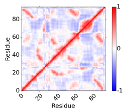	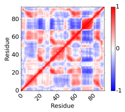	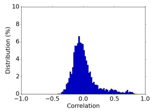	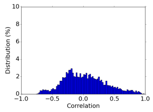
89	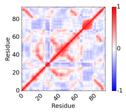	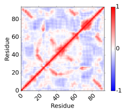	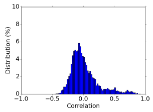	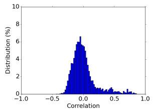
90	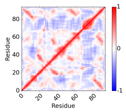	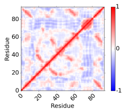	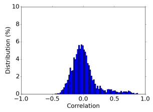	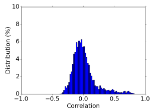
91	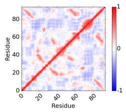	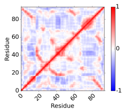	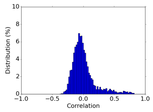	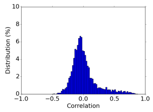
92	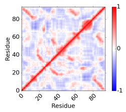	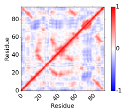	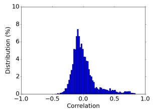	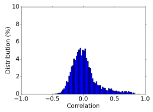
93	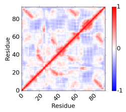	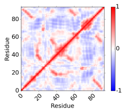	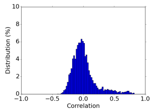	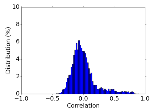
94	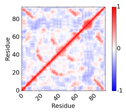	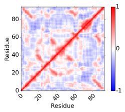	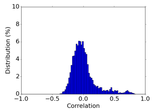	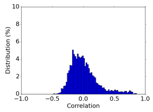

[a] In these heat maps, red means positive correlation, blue means negative correlation, white means no correlation.
[b] In the histogram plots, normalized distribution is plotted using 0.2 as bin width.
