# Supplementary material for: Rigid Residue Scan Simulations Systematically Reveal Residue Entropic Roles in Protein Allostery
Source: PLoS Comput Biol. 2016 Apr 26;12(4):e1004893. doi: 10.1371/journal.pcbi.1004893 (PMC4846164; doi:10.1371/journal.pcbi.1004893)
Supplement: S3 Table — (PDF) [file pcbi.1004893.s007.pdf]

Table S3: Relative entropies (ΔS) and differences ($\Delta\Delta S$) of PDZ2 between unbound and bound states. Entropy of PDZ2 from unperturbed unbound stimulation is set as reference value, and listed as zero. The Residue column represents simulations in which that particular residue is subjected to rigid body constraints. In Delta (Absolute) column, absolute difference between relative entropies of unbound and bound states are listed.

Residue	Unbound ΔS	Bound ΔS	Delta (Absolute) $\Delta\Delta S$
None	0.000	0.016	0.016
1	0.061	0.106	0.045
2	0.063	-0.065	0.128
3	0.171	0.033	0.138
4	0.118	0.094	0.024
5	-0.021	0.039	0.060
6	0.052	0.014	0.038
7	0.096	0.251	0.155
8	0.015	-0.044	0.059
9	0.107	0.037	0.070
10	0.028	0.208	0.180
11	0.022	-0.010	0.032
12	0.068	0.166	0.098
13	0.013	-0.052	0.065
14	0.071	0.057	0.014
15	0.084	0.083	0.001
16	0.015	-0.003	0.018
17	0.041	0.127	0.086
18	0.045	0.031	0.014
19	0.055	0.157	0.102
20	0.146	0.018	0.128
21	0.079	-0.022	0.101
22	0.145	0.010	0.135
23	0.031	0.102	0.071
24	0.118	0.040	0.078
25	0.106	0.136	0.030
26	0.099	0.139	0.040
27	0.068	-0.027	0.095
28	0.091	0.093	0.002
29	0.141	0.064	0.077
30	0.095	-0.013	0.108
31	0.024	0.016	0.008
32	0.060	0.021	0.039
33	0.074	0.228	0.154
34	0.168	0.039	0.129
35	-0.057	0.064	0.121
36	-0.018	-0.048	0.030
37	0.030	0.055	0.025
38	0.119	0.009	0.110
39	0.120	0.001	0.119
40	0.094	0.097	0.003
41	0.032	0.050	0.018
42	0.000	0.066	0.066
43	0.012	0.122	0.110
44	0.060	0.039	0.021

Table S3: Relative entropies (ΔS) and differences ($\Delta\Delta S$) of PDZ2 between unbound and bound states. Entropy of PDZ2 from unperturbed unbound stimulation is set as reference value, and listed as zero. The Residue column represents simulations in which that particular residue is subjected to rigid body constraints. In Delta (Absolute) column, absolute difference between relative entropies of unbound and bound states are listed.

Residue	Unbound ΔS	Bound ΔS	Delta (Absolute) $\Delta\Delta S$
45	0.067	0.041	0.026
46	0.120	0.146	0.026
47	0.044	-0.039	0.083
48	0.050	0.144	0.094
49	0.099	0.019	0.080
50	0.254	0.011	0.243
51	0.081	0.062	0.019
52	0.122	0.072	0.050
53	0.024	0.021	0.003
54	0.000	0.099	0.099
55	-0.020	0.060	0.080
56	0.093	0.118	0.025
57	-0.007	0.071	0.078
58	0.061	0.225	0.164
59	0.240	-0.020	0.260
60	0.073	0.020	0.053
61	-0.018	0.005	0.023
62	0.135	-0.041	0.176
63	0.010	-0.007	0.017
64	0.074	0.097	0.023
65	0.069	0.012	0.057
66	0.066	0.157	0.091
67	0.106	0.033	0.073
68	-0.003	0.194	0.197
69	0.022	-0.023	0.045
70	0.233	0.063	0.170
71	0.016	0.074	0.058
72	-0.100	0.070	0.170
73	0.015	0.111	0.096
74	0.015	0.062	0.047
75	0.011	0.052	0.041
76	0.126	0.079	0.047
77	0.073	0.049	0.024
78	0.010	0.020	0.010
79	-0.043	-0.041	0.002
80	0.092	0.059	0.033
81	0.039	0.043	0.004
82	0.014	0.069	0.055
83	-0.014	0.042	0.056
84	0.051	-0.015	0.066
85	0.067	0.017	0.050
86	0.006	-0.015	0.021
87	0.046	-0.051	0.097
88	0.064	0.405	0.341
89	0.015	0.122	0.107

Table S3: Relative entropies (ΔS) and differences ($\Delta\Delta S$) of PDZ2 between unbound and bound states. Entropy of PDZ2 from unperturbed unbound stimulation is set as reference value, and listed as zero. The Residue column represents simulations in which that particular residue is subjected to rigid body constraints. In Delta (Absolute) column, absolute difference between relative entropies of unbound and bound states are listed.

Residue	Unbound ΔS	Bound ΔS	Delta (Absolute) $\Delta\Delta S$
90	0.087	0.073	0.014
91	-0.033	0.031	0.064
92	0.027	0.133	0.106
93	0.068	0.090	0.022
94	-0.040	0.100	0.140
