# Supplementary material for: Rigid Residue Scan Simulations Systematically Reveal Residue Entropic Roles in Protein Allostery
Source: PLoS Comput Biol. 2016 Apr 26;12(4):e1004893. doi: 10.1371/journal.pcbi.1004893 (PMC4846164; doi:10.1371/journal.pcbi.1004893)
Supplement: S4 Table — (PDF) [file pcbi.1004893.s008.pdf]

Table S4: Relative entropies (ΔS) and differences ($\Delta\Delta S$) of PDZ2 between unbound and bound states sorted with ascending order. Each column from Table S3 is sorted separately, therefore with separate residue list.

	Rigid Unbound		Rigid Bound		Delta Entropy(Absolute)	
#	Residue	ΔS	Residue	ΔS	Residue	$\Delta\Delta S$
1	72	-0.100	2	-0.065	15	0.001
2	35	-0.057	13	-0.052	28	0.002
3	79	-0.043	87	-0.051	79	0.002
4	94	-0.040	36	-0.048	40	0.003
5	91	-0.033	8	-0.044	53	0.003
6	5	-0.021	62	-0.041	81	0.004
7	55	-0.020	79	-0.041	31	0.008
8	61	-0.018	47	-0.039	78	0.010
9	36	-0.018	27	-0.027	90	0.014
10	83	-0.014	69	-0.023	14	0.014
11	57	-0.007	21	-0.022	18	0.014
12	68	-0.003	59	-0.020	0	0.016
13	54	0.000	84	-0.015	63	0.017
14	42	0.000	86	-0.015	41	0.018
15	0	0.000	30	-0.013	16	0.018
16	86	0.006	11	-0.010	51	0.019
17	63	0.010	63	-0.007	86	0.021
18	78	0.010	16	-0.003	44	0.021
19	75	0.011	39	0.001	93	0.022
20	43	0.012	61	0.005	61	0.023
21	13	0.013	38	0.009	64	0.023
22	82	0.014	22	0.010	4	0.024
23	73	0.015	50	0.011	77	0.024
24	74	0.015	65	0.012	37	0.025
25	89	0.015	6	0.014	56	0.025
26	16	0.015	31	0.016	45	0.026
27	8	0.015	0	0.016	46	0.026
28	71	0.016	85	0.017	36	0.030
29	11	0.022	20	0.018	25	0.030
30	69	0.022	49	0.019	11	0.032
31	53	0.024	78	0.020	80	0.033
32	31	0.024	60	0.020	6	0.038
33	92	0.027	32	0.021	32	0.039
34	10	0.028	53	0.021	26	0.040
35	37	0.030	91	0.031	75	0.041
36	23	0.031	18	0.031	1	0.045
37	41	0.032	67	0.033	69	0.045
38	81	0.039	3	0.033	76	0.047
39	17	0.041	9	0.037	74	0.047
40	47	0.044	44	0.039	85	0.050
41	18	0.045	5	0.039	52	0.050
42	87	0.046	34	0.039	60	0.053
43	48	0.050	24	0.040	82	0.055
44	84	0.051	45	0.041	83	0.056
45	6	0.052	83	0.042	65	0.057
46	19	0.055	81	0.043	71	0.058
47	44	0.060	77	0.049	8	0.059
48	32	0.060	41	0.050	5	0.060

Table S4: Relative entropies (ΔS) and differences ($\Delta\Delta S$) of PDZ2 between unbound and bound states sorted with ascending order. Each column from Table S3 is sorted separately, therefore with separate residue list.

	Rigid Unbound		Rigid Bound		Delta Entropy(Absolute)	
#	Residue	ΔS	Residue	ΔS	Residue	$\Delta\Delta S$
49	1	0.061	75	0.052	91	0.064
50	58	0.061	37	0.055	13	0.065
51	2	0.063	14	0.057	84	0.066
52	88	0.064	80	0.059	42	0.066
53	66	0.066	55	0.060	9	0.070
54	85	0.067	74	0.062	23	0.071
55	45	0.067	51	0.062	67	0.073
56	27	0.068	70	0.063	29	0.077
57	93	0.068	35	0.064	57	0.078
58	12	0.068	29	0.064	24	0.078
59	65	0.069	42	0.066	55	0.080
60	14	0.071	82	0.069	49	0.080
61	77	0.073	72	0.070	47	0.083
62	60	0.073	57	0.071	17	0.086
63	64	0.074	52	0.072	66	0.091
64	33	0.074	90	0.073	48	0.094
65	21	0.079	71	0.074	27	0.095
66	51	0.081	76	0.079	73	0.096
67	15	0.084	15	0.083	87	0.097
68	90	0.087	93	0.090	12	0.098
69	28	0.091	28	0.093	54	0.099
70	80	0.092	4	0.094	21	0.101
71	56	0.093	64	0.097	19	0.102
72	40	0.094	40	0.097	92	0.106
73	30	0.095	54	0.099	89	0.107
74	7	0.096	94	0.100	30	0.108
75	49	0.099	23	0.102	43	0.110
76	26	0.099	1	0.106	38	0.110
77	67	0.106	73	0.111	39	0.119
78	25	0.106	56	0.118	35	0.121
79	9	0.107	89	0.122	2	0.128
80	4	0.118	43	0.122	20	0.128
81	24	0.118	17	0.127	34	0.129
82	38	0.119	92	0.133	22	0.135
83	46	0.120	25	0.136	3	0.138
84	39	0.120	26	0.139	94	0.140
85	52	0.122	48	0.144	33	0.154
86	76	0.126	46	0.146	7	0.155
87	62	0.135	19	0.157	58	0.164
88	29	0.141	66	0.157	72	0.170
89	22	0.145	12	0.166	70	0.170
90	20	0.146	68	0.194	62	0.176
91	34	0.168	10	0.208	10	0.180
92	3	0.171	58	0.225	68	0.197
93	70	0.233	33	0.228	50	0.243
94	59	0.240	7	0.251	59	0.260
95	50	0.254	88	0.405	88	0.341
