# Supplementary material for: Rigid Residue Scan Simulations Systematically Reveal Residue Entropic Roles in Protein Allostery
Source: PLoS Comput Biol. 2016 Apr 26;12(4):e1004893. doi: 10.1371/journal.pcbi.1004893 (PMC4846164; doi:10.1371/journal.pcbi.1004893)
Supplement: S5 Table — (PDF) [file pcbi.1004893.s009.pdf]

Table S5: Average entropic response of individual residues upon rigid body perturbations. Each residue number represents particular residue, not simulation.

Residue	Rigid Unbound ΔS	Rigid Bound ΔS
1	-0.0154	0.0007
2	-0.0167	0.0037
3	-0.0010	0.0012
4	0.0001	0.0003
5	-0.0006	0.0008
6	-0.0068	0.0033
7	0.0041	0.0052
8	-0.0010	0.0043
9	0.0036	0.0039
10	0.0037	0.0001
11	-0.0048	0.0051
12	0.0009	0.0023
13	0.0050	0.0084
14	0.0024	-0.0021
15	0.0001	0.0002
16	0.0018	0.0007
17	0.0030	0.0012
18	0.0104	-0.0038
19	0.0006	0.0007
20	-0.0083	-0.0079
21	-0.0005	-0.0019
22	0.0089	0.0183
23	0.0019	0.0064
24	-0.0002	-0.0016
25	0.0007	-0.0003
26	0.0097	0.0067
27	0.0061	0.0002
28	0.0160	-0.0053
29	0.0059	-0.0022
30	0.0030	-0.0003
31	0.0352	0.0001
32	-0.0007	-0.0061
33	0.0025	0.0002
34	0.0013	-0.0006
35	0.0025	0.0009
36	0.0004	-0.0015
37	0.0013	-0.0023
38	0.0008	-0.0161
39	0.0014	-0.0016
40	0.0049	-0.0046
41	0.0016	-0.0016
42	0.0013	-0.0010
43	-0.0000	0.0004
44	0.0008	0.0003
45	0.0020	0.0006
46	0.0018	0.0003
47	0.0013	0.0018
48	0.0007	-0.0009
49	0.0056	0.0022

Table S5: Average entropic response of individual residues upon rigid body perturbations. Each residue number represents particular residue, not simulation.

Residue	Rigid Unbound ΔS	Rigid Bound ΔS
50	0.0016	0.0006
51	0.0022	0.0023
52	-0.0044	0.0131
53	-0.0108	-0.0124
54	0.0070	-0.0004
55	0.0011	0.0003
56	0.0021	-0.0015
57	0.0002	-0.0049
58	0.0014	0.0001
59	0.0055	0.0049
60	0.0005	0.0001
61	0.0107	0.0079
62	-0.0006	0.0035
63	0.0001	0.0001
64	-0.0067	-0.0014
65	0.0052	-0.0011
66	-0.0025	-0.0075
67	-0.0025	-0.0037
68	0.0013	-0.0002
69	0.0017	0.0001
70	0.0026	0.0011
71	-0.0016	-0.0028
72	0.0006	0.0021
73	-0.0005	0.0004
74	0.0014	0.0007
75	0.0001	0.0084
76	-0.0051	0.0015
77	0.0014	-0.0079
78	0.0018	-0.0030
79	-0.0098	-0.0058
80	-0.0050	0.0032
81	-0.0005	0.0046
82	0.0006	0.0016
83	0.0008	0.0030
84	0.0091	0.0029
85	0.0023	0.0148
86	-0.0027	0.0022
87	-0.0048	0.0041
88	0.0043	0.0027
89	0.0040	0.0083
90	-0.0004	-0.0036
91	0.0070	-0.0030
92	0.0019	-0.0016
93	0.0051	-0.0108
94	-0.0018	-0.0011
