# Supplementary material for: Rigid Residue Scan Simulations Systematically Reveal Residue Entropic Roles in Protein Allostery
Source: PLoS Comput Biol. 2016 Apr 26;12(4):e1004893. doi: 10.1371/journal.pcbi.1004893 (PMC4846164; doi:10.1371/journal.pcbi.1004893)
Supplement: S6 Table — (PDF) [file pcbi.1004893.s010.pdf]

Table S6: Average entropic response of individual residues upon rigid body perturbations sorted with descending order. Each column from Table S5 is sorted separately, therefore with separate Residue list.

	Unbound Residue Entropy		Bound Residue Entropy	
#	Residue	ΔS	Residue	ΔS
1	31	0.0352	22	0.0183
2	28	0.0160	85	0.0148
3	61	0.0107	52	0.0131
4	18	0.0104	75	0.0084
5	26	0.0097	13	0.0084
6	84	0.0091	89	0.0083
7	22	0.0089	61	0.0079
8	54	0.0070	26	0.0067
9	91	0.0070	23	0.0064
10	27	0.0061	7	0.0052
11	29	0.0059	11	0.0051
12	49	0.0056	59	0.0049
13	59	0.0055	81	0.0046
14	65	0.0052	8	0.0043
15	93	0.0051	87	0.0041
16	13	0.0050	9	0.0039
17	40	0.0049	2	0.0037
18	88	0.0043	62	0.0035
19	7	0.0041	6	0.0033
20	89	0.0040	80	0.0032
21	10	0.0037	83	0.0030
22	9	0.0036	84	0.0029
23	17	0.0030	88	0.0027
24	30	0.0030	51	0.0023
25	70	0.0026	12	0.0023
26	35	0.0025	49	0.0022
27	33	0.0025	86	0.0022
28	14	0.0024	72	0.0021
29	85	0.0023	47	0.0018
30	51	0.0022	82	0.0016
31	56	0.0021	76	0.0015
32	45	0.0020	17	0.0012
33	92	0.0019	3	0.0012
34	23	0.0019	70	0.0011
35	78	0.0018	35	0.0009
36	16	0.0018	5	0.0008
37	46	0.0018	19	0.0007
38	69	0.0017	1	0.0007
39	50	0.0016	74	0.0007
40	41	0.0016	16	0.0007
41	58	0.0014	50	0.0006
42	77	0.0014	45	0.0006
43	74	0.0014	73	0.0004
44	39	0.0014	43	0.0004
45	37	0.0013	46	0.0003
46	68	0.0013	4	0.0003
47	34	0.0013	55	0.0003
48	47	0.0013	44	0.0003

Table S6: Average entropic response of individual residues upon rigid body perturbations sorted with descending order. Each column from Table S5 is sorted separately, therefore with separate Residue list.

	Unbound Residue Entropy		Bound Residue Entropy	
#	Residue	ΔS	Residue	ΔS
49	42	0.0013	27	0.0002
50	55	0.0011	15	0.0002
51	12	0.0009	33	0.0002
52	38	0.0008	58	0.0001
53	44	0.0008	69	0.0001
54	83	0.0008	31	0.0001
55	48	0.0007	60	0.0001
56	25	0.0007	63	0.0001
57	19	0.0006	10	0.0001
58	82	0.0006	68	-0.0002
59	72	0.0006	25	-0.0003
60	60	0.0005	30	-0.0003
61	36	0.0004	54	-0.0004
62	57	0.0002	34	-0.0006
63	63	0.0001	48	-0.0009
64	75	0.0001	42	-0.0010
65	4	0.0001	65	-0.0011
66	15	0.0001	94	-0.0011
67	43	-0.0000	64	-0.0014
68	24	-0.0002	56	-0.0015
69	90	-0.0004	36	-0.0015
70	21	-0.0005	24	-0.0016
71	73	-0.0005	92	-0.0016
72	81	-0.0005	39	-0.0016
73	62	-0.0006	41	-0.0016
74	5	-0.0006	21	-0.0019
75	32	-0.0007	14	-0.0021
76	8	-0.0010	29	-0.0022
77	3	-0.0010	37	-0.0023
78	71	-0.0016	71	-0.0028
79	94	-0.0018	78	-0.0030
80	67	-0.0025	91	-0.0030
81	66	-0.0025	90	-0.0036
82	86	-0.0027	67	-0.0037
83	52	-0.0044	18	-0.0038
84	87	-0.0048	40	-0.0046
85	11	-0.0048	57	-0.0049
86	80	-0.0050	28	-0.0053
87	76	-0.0051	79	-0.0058
88	64	-0.0067	32	-0.0061
89	6	-0.0068	66	-0.0075
90	20	-0.0083	20	-0.0079
91	79	-0.0098	77	-0.0079
92	53	-0.0108	93	-0.0108
93	1	-0.0154	53	-0.0124
94	2	-0.0167	38	-0.0161
