# Supplementary material for: Rigid Residue Scan Simulations Systematically Reveal Residue Entropic Roles in Protein Allostery
Source: PLoS Comput Biol. 2016 Apr 26;12(4):e1004893. doi: 10.1371/journal.pcbi.1004893 (PMC4846164; doi:10.1371/journal.pcbi.1004893)
Supplement: S7 Table — (PDF) [file pcbi.1004893.s011.pdf]

Table S7: Dot products of five lowest frequency quasi-harmonic modes (PC1-PC5) from seven sets of 30 ns trajectories with the PC1 to PC5 from whole 210 ns trajectory

Unperturbed Unbound State

30ns trajectory set	PC1	PC2	PC3	PC4	PC5
1	0.961	-0.747	0.256	-0.463	0.477
2	0.938	0.467	-0.204	0.170	0.471
3	-0.922	0.417	0.098	-0.062	-0.212
4	0.964	0.505	0.136	0.115	-0.137
5	0.924	-0.164	0.048	0.313	0.005
6	0.828	-0.376	-0.175	0.187	0.481
7	0.963	-0.277	0.432	0.131	0.226
Average unsigned mean	0.929	0.422	0.193	0.206	0.287
Standard deviation	0.044	0.171	0.116	0.128	0.177

Unperturbed Bound State

30ns trajectory set	PC1	PC2	PC3	PC4	PC5
1	0.917	-0.415	0.437	-0.013	0.246
2	0.920	-0.418	0.169	-0.299	0.473
3	0.941	0.668	0.074	0.140	-0.063
4	0.955	-0.742	-0.151	-0.132	0.004
5	0.916	0.012	0.057	-0.088	-0.489
6	0.825	0.354	0.431	-0.288	-0.332
7	0.818	0.606	0.110	-0.273	0.172
Average unsigned mean	0.899	0.459	0.204	0.176	0.254
Standard deviation	0.051	0.227	0.150	0.103	0.175
