# Supplementary material for: Rigid Residue Scan Simulations Systematically Reveal Residue Entropic Roles in Protein Allostery
Source: PLoS Comput Biol. 2016 Apr 26;12(4):e1004893. doi: 10.1371/journal.pcbi.1004893 (PMC4846164; doi:10.1371/journal.pcbi.1004893)
Supplement: S8 Table — (PDF) [file pcbi.1004893.s012.pdf]

Table S8: Dot products among PC1 modes from seven 30 ns trajectories in both unperturbed unbound state and unperturbed bound state.

Unperturbed Unbound State

30ns trajectory set	1	2	3	4	5	6	7
1	1.000	0.926	-0.921	0.916	0.834	0.753	0.930
2	0.926	1.000	-0.960	0.862	0.814	0.722	0.858
3	-0.921	-0.960	1.000	-0.856	-0.776	-0.702	-0.847
4	0.916	0.862	-0.856	1.000	0.897	0.779	0.934
5	0.834	0.814	-0.776	0.897	1.000	0.694	0.867
6	0.753	0.722	-0.702	0.779	0.694	1.000	0.834
7	0.930	0.858	-0.847	0.934	0.867	0.834	1.000
average values	0.842						
standard deviation	0.078						

Unperturbed Bound State

30ns trajectory set	1	2	3	4	5	6	7
1	1.000	0.903	0.864	0.837	0.790	0.722	0.675
2	0.903	1.000	0.868	0.819	0.761	0.744	0.728
3	0.864	0.868	1.000	0.919	0.825	0.717	0.672
4	0.837	0.819	0.919	1.000	0.938	0.696	0.705
5	0.790	0.761	0.825	0.938	1.000	0.653	0.680
6	0.722	0.744	0.717	0.696	0.653	1.000	0.887
7	0.675	0.728	0.672	0.705	0.680	0.887	1.000
average values	0.781						
standard deviation	0.089						
