# Supplementary material for: Rigid Residue Scan Simulations Systematically Reveal Residue Entropic Roles in Protein Allostery
Source: PLoS Comput Biol. 2016 Apr 26;12(4):e1004893. doi: 10.1371/journal.pcbi.1004893 (PMC4846164; doi:10.1371/journal.pcbi.1004893)
Supplement: S9 Table — (PDF) [file pcbi.1004893.s013.pdf]

Table S9: Projections of simulations onto 2D-surface using two PC1 modes from unperturbed unbound and bound states. Unbound (green) and bound (brown) states are plotted on the same surface for comparison. Index indicates the residue being held rigid.

Residue	Projection	Residue	Projection
None	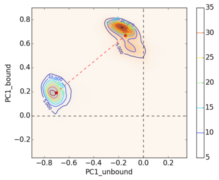		
1	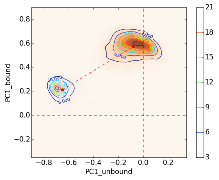	2	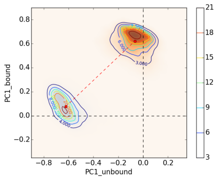
3	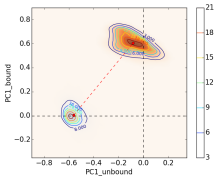	4	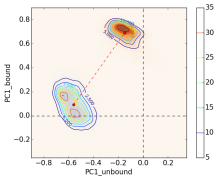
5	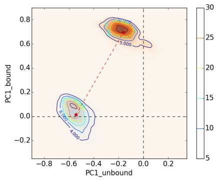	6	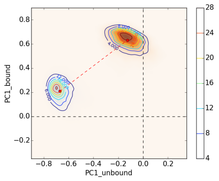
7	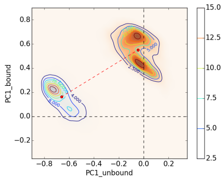	8	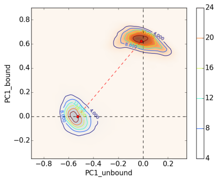
9	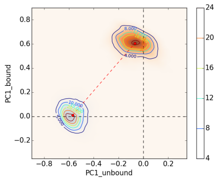	10	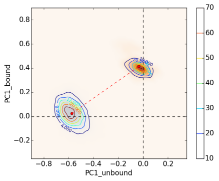
11	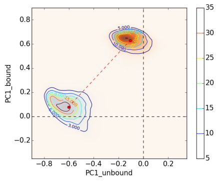	12	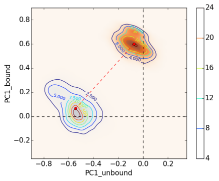

Table S9: Projections of simulations onto 2D-surface using two PC1 modes from unperturbed unbound and bound states. Unbound (green) and bound (brown) states are plotted on the same surface for comparison. Index indicates the residue being held rigid.

Residue	Projection	Residue	Projection
13	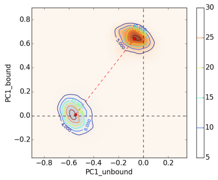	14	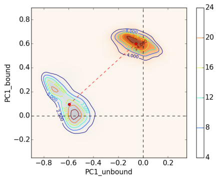
15	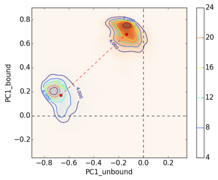	16	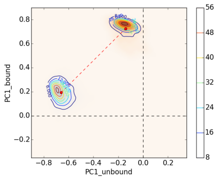
17	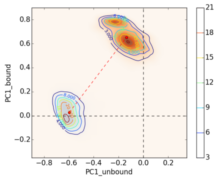	18	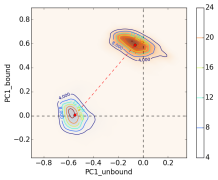
19	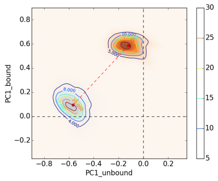	20	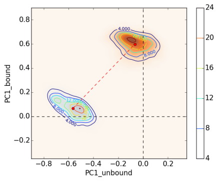
21	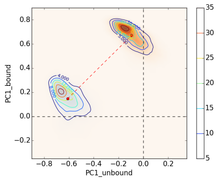	22	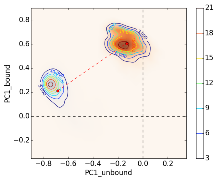
23	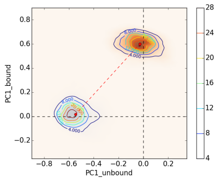	24	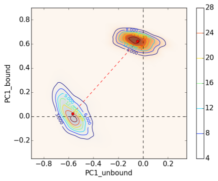
25	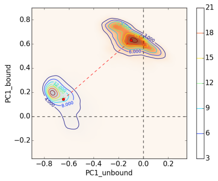	26	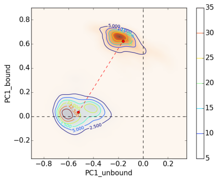

Table S9: Projections of simulations onto 2D-surface using two PC1 modes from unperturbed unbound and bound states. Unbound (green) and bound (brown) states are plotted on the same surface for comparison. Index indicates the residue being held rigid.

Residue	Projection	Residue	Projection
27	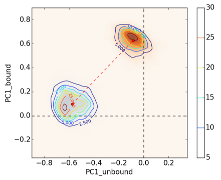	28	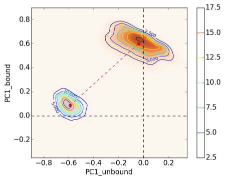
29	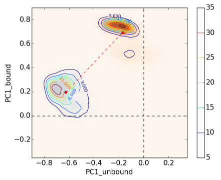	30	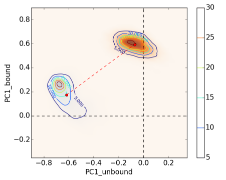
31	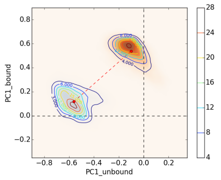	32	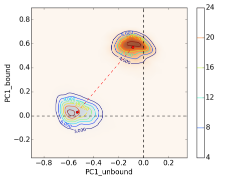
33	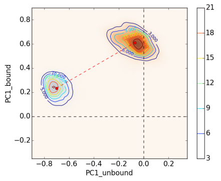	34	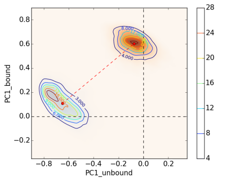
35	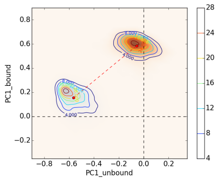	36	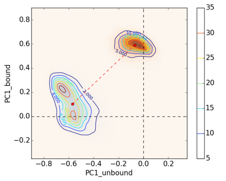
37	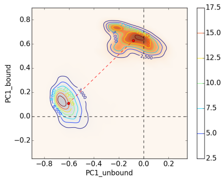	38	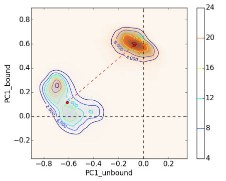
39	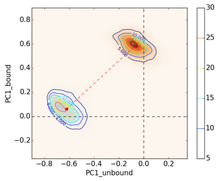	40	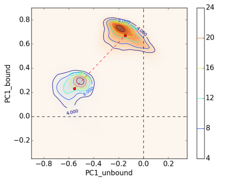

Table S9: Projections of simulations onto 2D-surface using two PC1 modes from unperturbed unbound and bound states. Unbound (green) and bound (brown) states are plotted on the same surface for comparison. Index indicates the residue being held rigid.

Residue	Projection	Residue	Projection
41	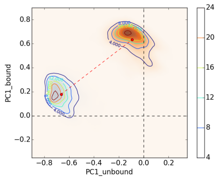	42	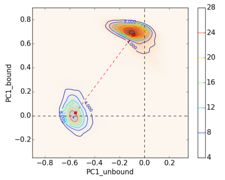
43	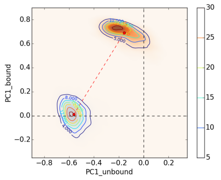	44	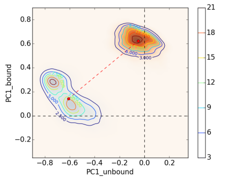
45	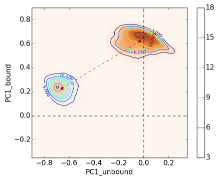	46	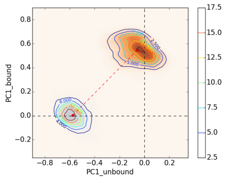
47	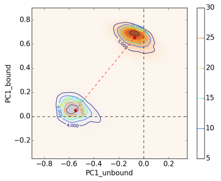	48	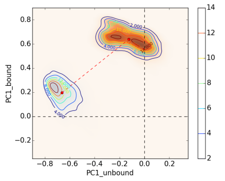
49	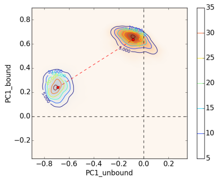	50	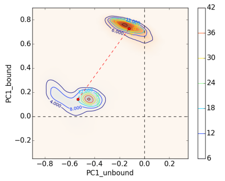
51	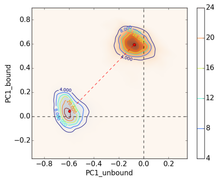	52	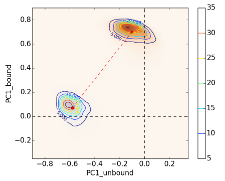
53	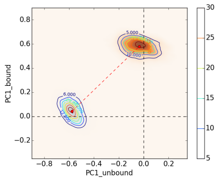	54	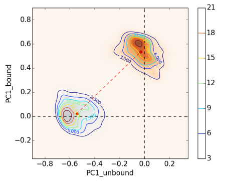

Table S9: Projections of simulations onto 2D-surface using two PC1 modes from unperturbed unbound and bound states. Unbound (green) and bound (brown) states are plotted on the same surface for comparison. Index indicates the residue being held rigid.

Residue	Projection	Residue	Projection
55	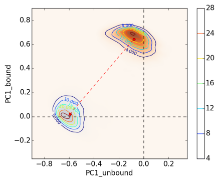	56	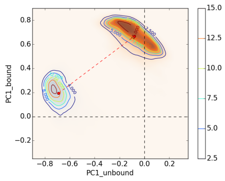
57	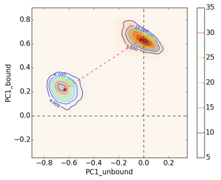	58	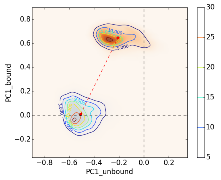
59	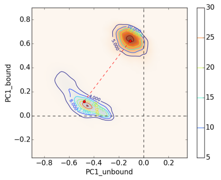	60	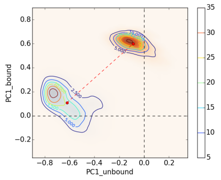
61	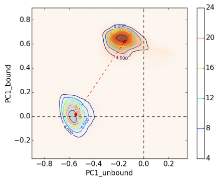	62	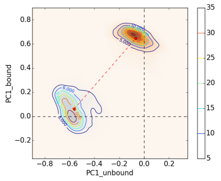
63	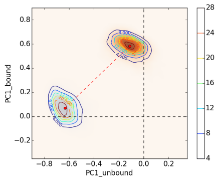	64	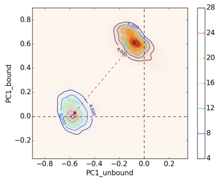
65	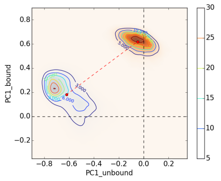	66	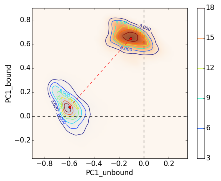
67	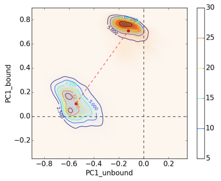	68	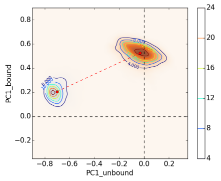

Table S9: Projections of simulations onto 2D-surface using two PC1 modes from unperturbed unbound and bound states. Unbound (green) and bound (brown) states are plotted on the same surface for comparison. Index indicates the residue being held rigid.

Residue	Projection	Residue	Projection
69	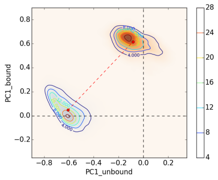	70	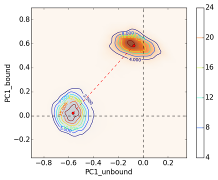
71	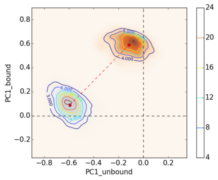	72	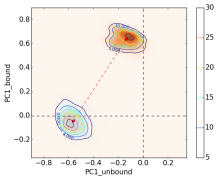
73	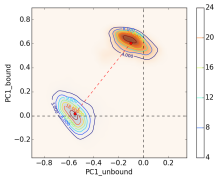	74	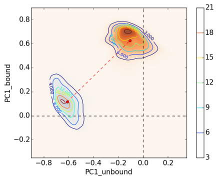
75	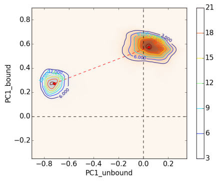	76	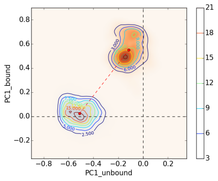
77	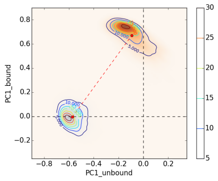	78	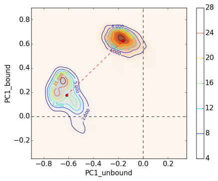
79	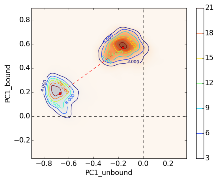	80	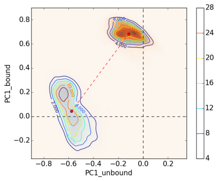
81	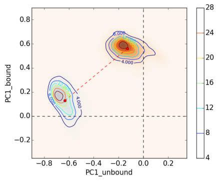	82	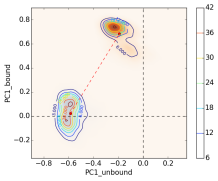

Table S9: Projections of simulations onto 2D-surface using two PC1 modes from unperturbed unbound and bound states. Unbound (green) and bound (brown) states are plotted on the same surface for comparison. Index indicates the residue being held rigid.

Residue	Projection	Residue	Projection
83	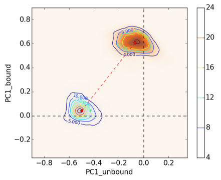	84	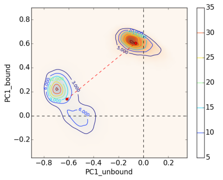
85	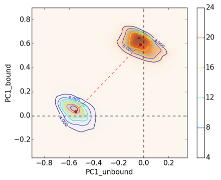	86	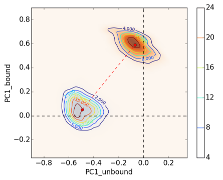
87	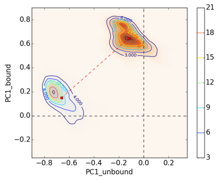	88	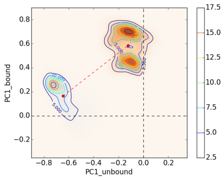
89	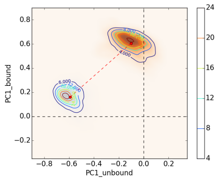	90	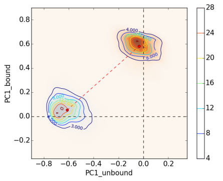
91	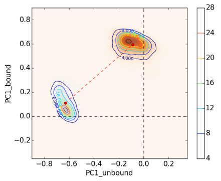	92	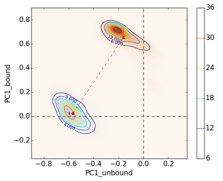
93	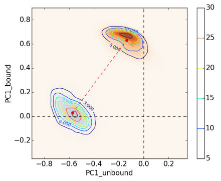	94	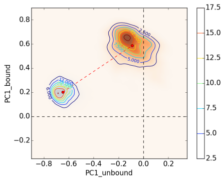
