# Supplementary material for: Rigid Residue Scan Simulations Systematically Reveal Residue Entropic Roles in Protein Allostery
Source: PLoS Comput Biol. 2016 Apr 26;12(4):e1004893. doi: 10.1371/journal.pcbi.1004893 (PMC4846164; doi:10.1371/journal.pcbi.1004893)
Supplement: S10 Table — (PDF) [file pcbi.1004893.s014.pdf]

Table S10: Average distance between distributions of unbound and bound states projected onto 2D-surface using two PC1 modes. Simulation index indicates the residue being held rigid.

Simulation	Distance ($\mathring{A}$)
0	0.734
1	0.675
2	0.779
3	0.768
4	0.725
5	0.782
6	0.689
7	0.728
8	0.810
9	0.780
10	0.665
11	0.743
12	0.712
13	0.791
14	0.736
15	0.734
16	0.742
17	0.775
18	0.758
19	0.665
20	0.728
21	0.734
22	0.680
23	0.783
24	0.795
25	0.735
26	0.690
27	0.720
28	0.754
29	0.675
30	0.689
31	0.622
32	0.702
33	0.733
34	0.759
35	0.665
36	0.697
37	0.737
38	0.714
39	0.762
40	0.598
41	0.729
42	0.803
43	0.793
44	0.739
45	0.739
46	0.748
47	0.768
48	0.695

Table S10: Average distance between distributions of unbound and bound states projected onto 2D-surface using two PC1 modes. Simulation index indicates the residue being held rigid.

Simulation	Distance ($\AA$)
49	0.722
50	0.716
51	0.759
52	0.787
53	0.762
54	0.725
55	0.808
56	0.772
57	0.741
58	0.701
59	0.626
60	0.725
61	0.722
62	0.766
63	0.731
64	0.752
65	0.721
66	0.754
67	0.736
68	0.742
69	0.773
70	0.745
71	0.694
72	0.804
73	0.744
74	0.718
75	0.819
76	0.659
77	0.828
78	0.637
79	0.634
80	0.788
81	0.663
82	0.769
83	0.709
84	0.722
85	0.763
86	0.684
87	0.734
88	0.669
89	0.662
90	0.784
91	0.726
92	0.751
93	0.741
94	0.679
