# Supplementary material for: Rigid Residue Scan Simulations Systematically Reveal Residue Entropic Roles in Protein Allostery
Source: PLoS Comput Biol. 2016 Apr 26;12(4):e1004893. doi: 10.1371/journal.pcbi.1004893 (PMC4846164; doi:10.1371/journal.pcbi.1004893)
Supplement: S11 Table — (PDF) [file pcbi.1004893.s015.pdf]

Table S11: Average distance between distributions of unbound and bound states projected onto 2D-surface using two PC1 modes sorted with ascending order.

Rank	Simulation	Distance ($\mathring{A}$)
1	40	0.598
2	31	0.622
3	59	0.626
4	79	0.634
5	78	0.637
6	76	0.659
7	89	0.662
8	81	0.663
9	35	0.665
10	19	0.665
11	10	0.665
12	88	0.669
13	1	0.675
14	29	0.675
15	94	0.679
16	22	0.680
17	86	0.684
18	30	0.689
19	6	0.689
20	26	0.690
21	71	0.694
22	48	0.695
23	36	0.697
24	58	0.701
25	32	0.702
26	83	0.709
27	12	0.712
28	38	0.714
29	50	0.716
30	74	0.718
31	27	0.720
32	65	0.721
33	84	0.722
34	49	0.722
35	61	0.722
36	4	0.725
37	60	0.725
38	54	0.725
39	91	0.726
40	7	0.728
41	20	0.728
42	41	0.729
43	63	0.731
44	33	0.733
45	87	0.734
46	0	0.734
47	15	0.734
48	21	0.734
49	25	0.735

Table S11: Average distance between distributions of unbound and bound states projected onto 2D-surface using two PC1 modes sorted with ascending order.

Rank	Simulation	Distance ($\AA$)
50	14	0.736
51	67	0.736
52	37	0.737
53	44	0.739
54	45	0.739
55	93	0.741
56	57	0.741
57	16	0.742
58	68	0.742
59	11	0.743
60	73	0.744
61	70	0.745
62	46	0.748
63	92	0.751
64	64	0.752
65	66	0.754
66	28	0.754
67	18	0.758
68	34	0.759
69	51	0.759
70	39	0.762
71	53	0.762
72	85	0.763
73	62	0.766
74	3	0.768
75	47	0.768
76	82	0.769
77	56	0.772
78	69	0.773
79	17	0.775
80	2	0.779
81	9	0.780
82	5	0.782
83	23	0.783
84	90	0.784
85	52	0.787
86	80	0.788
87	13	0.791
88	43	0.793
89	24	0.795
90	42	0.803
91	72	0.804
92	55	0.808
93	8	0.810
94	75	0.819
95	77	0.828
